# Cell-based versus corticosteroid injections for knee pain in osteoarthritis: a randomized phase 3 trial

**DOI:** 10.1038/s41591-023-02632-w

**Published:** 2023-11-02

**Authors:** Ken Mautner, Michael Gottschalk, Scott D. Boden, Alison Akard, Won C. Bae, Lora Black, Blake Boggess, Paramita Chatterjee, Christine B. Chung, Kirk A. Easley, Greg Gibson, Josh Hackel, Katie Jensen, Linda Kippner, Chad Kurtenbach, Joanne Kurtzberg, R. Amadeus Mason, Benjamin Noonan, Krishnendu Roy, Verle Valentine, Carolyn Yeago, Hicham Drissi

**Affiliations:** 1 Department of Orthopaedics, Emory University, Atlanta, GA USA; 2 Department of Radiology, University of California, Davis, La Jolla, CA USA; 3 Sanford Health, Sioux Falls, SD USA; 4 Duke Sports Medicine, Durham, NC USA; 5 Marcus Center for Therapeutic Cell Characterization and Manufacturing, Georgia Institute of Technology, Atlanta, GA USA; 6 Department of Biostatistics and Bioinformatics, Emory University, Atlanta, GA USA; 7 Center for Integrative Genomics, Georgia Institute of Technology, Atlanta, GA USA; 8 Andrews Institute, Gulf Breeze, AL USA; 9 Marcus Center for Therapeutic Cures, Duke University, Durham, NC USA; 10 Sanford Health, Fargo, ND USA; 11 Biomedical Engineering, Vanderbilt University, Nashville, TN USA

**Keywords:** Mesenchymal stem cells, Stem-cell research

## Abstract

Various types of cellular injection have become a popular and costly treatment option for patients with knee osteoarthritis despite a paucity of literature establishing relative efficacy to each other or corticosteroid injections. Here we aimed to identify the safety and efficacy of cell injections from autologous bone marrow aspirate concentrate, autologous adipose stromal vascular fraction and allogeneic human umbilical cord tissue-derived mesenchymal stromal cells, in comparison to corticosteroid injection (CSI). The study was a phase 2/3, four-arm parallel, multicenter, single-blind, randomized, controlled clinical trial with 480 patients with a diagnosis of knee osteoarthritis (Kellgren–Lawrence II–IV). Participants were randomized to the three different arms with a 3:1 distribution. Arm 1: autologous bone marrow aspirate concentrate (*n* = 120), CSI (*n* = 40); arm 2: umbilical cord tissue-derived mesenchymal stromal cells (*n* = 120), CSI (*n* = 40); arm 3: stromal vascular fraction (*n* = 120), CSI (*n* = 40). The co-primary endpoints were the visual analog scale pain score and Knee injury and Osteoarthritis Outcome Score pain score at 12 months versus baseline. Analyses of our primary endpoints, with 440 patients, revealed that at 1 year post injection, none of the three orthobiologic injections was superior to another, or to the CSI control. In addition, none of the four groups showed a significant change in magnetic resonance imaging osteoarthritis score compared to baseline. No procedure-related serious adverse events were reported during the study period. In summary, this study shows that at 1 year post injection, there was no superior orthobiologic as compared to CSI for knee osteoarthritis. ClinicalTrials.gov Identifier: NCT03818737

## Abstract (web summary)

The multicenter phase 3 trial of stem cell therapy for osteoarthritis (MILES) for knee pain revealed that cell therapies showed no significant difference in knee pain outcomes, compared to corticosteroid injections, 1 year following treatment.

## Main

Osteoarthritis (OA) is a degenerative condition that affects millions of patients every year. Although arthritis is considered a disease of abnormal joint mechanics marked by periods of inflammation, the underlying etiology is biochemically mediated leading to destruction of articular cartilage^1^. OA presents a clinical dilemma in the United States and around the world, affecting more than 54 million Americans according to the 2017 reported assessment by Barbour et al.^2^. The economic burden and cost of care for treatment and lost productivity for 54 million Americans caused by OA amounts to US$60 billion annually in the United States, with recent studies suggesting health care costs alone between US$5.7 and US$15 billion^3,4^. Despite advances in diagnosis, medications and injections that control pain and inflammation in the short term, the quest for the development of a disease-modifying OA drug has proven unsuccessful.

The potential of mesenchymal stem cells (MSCs, also referred to as mesenchymal stromal cells) as a treatment for chronic diseases has been investigated in several areas of medicine, including orthopedics. Although still debatable, it is thought that common responses from these cells are inhibiting inflammation, protecting, and supporting chondrocytes, and providing a healthier joint environment^5–8^. In orthopedic practice, autologous cellular injections are widely used with the hopes of reducing pain and improving function. However, a thorough search of the orthopedic literature yielded limited injectable cell data, especially with well-designed randomized controlled trials^8–14^. Thus, a larger clinical trial could add information as to whether cellular treatments, while more expensive than corticosteroids, are more beneficial and if one cell therapy source outperforms another.

In this Article, our objective is to identify the most effective source of cellular injections for knee OA. To accomplish this, three types of cellular preparation including autologous bone marrow aspirate concentrate (BMAC), autologous stromal vascular fraction (SVF) and allogenic human umbilical cord tissue MSCs (UCT) were each compared against the gold-standard corticosteroid injection (CSI). Our co-primary outcome measures are visual analog scale (VAS) and Knee injury and Osteoarthritis Outcome Score (KOOS) pain from baseline to 1 year. We hypothesized that cell therapies would be superior to corticosteroids for treatment of knee OA at 1 year.

## Results

**Fig. 1:**
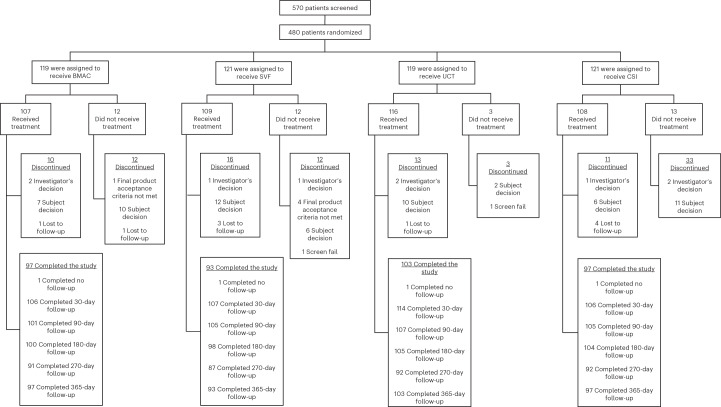
Consort diagram. Number randomized to each arm of study with dropouts and reason for dropout included.

Between March 2019 and June 2021, following prescreening by a research coordinator, 570 patients were screened, 480 of whom fulfilled eligibility criteria (84%) and were randomized into one of four cohorts and then three subsequent arms (see Fig. 1). All treatments exceeded minimally clinically important difference for both co-primary endpoints and sustained at 1 year post injection procedure. Table 1 provides the participants demographics for the study that were similar between the four treatment groups. Average age of patients in the overall study was 58.3 years with average body mass index (BMI) of 30.8 kg m^−2^. There were 214 (45.1%) males and 261 (54.9%) females who received injections. Although initially planned as a subgroup analysis, racial diversity was too small to perform statistical analysis. The breakdown of race by group was as follows. In the BMAC cohort there were zero Hispanic, 1 American Indian, 4 Asian, zero native Hawaiian, 19 African American, 92 white and 2 of unknown race (for example, multiple races). In the SVF cohort there were 5 Hispanic, 1 American Indian, 3 Asian, zero native Hawaiian, 15 African American, 99 white and 1 of unknown race. In the UCT cohort there were 5 Hispanic, zero American Indian, 4 Asian, zero native Hawaiian, 11 African American, 102 white and 1 of unknown race. In the CSI cohort there were 6 Hispanic, zero American Indian, two Asian, zero native Hawaiian, 12 African American, 104 white and 2 of unknown race.

**Table 1:** Demographic and Baseline Characteristics by Treatment Group for the Intent-to-Treat Population

	BMAC	SVF	UCT	CSI	All subjects
	*n* = 118	*n* = 119	*n* = 118	*n* = 120	*n* = 475
Age (years)					
Mean ± s.d.	58.6 ± 7.3	58.2 ± 7.3	57.9 ± 8.2	58.3 ± 8.1	58.3 ± 7.7
≥ 60, *n* (%)	61 (52%)	56 (47%)	54 (46%)	58 (48%)	229 (48%)
< 60, *n* (%)	57 (48%)	63 (53%)	64 (54%)	62 (52%)	246 (52%)
BMI (kg m^−2^), *n*	104	106	115	107	432
Mean ± s.d.	30.6 ± 6.0	30.5 ± 6.4	30.9 ± 5.4	31.2 ± 6.2	30.8 ± 6.0
KL grade, *n* (%)					
Grade 2	31 (26%)	34 (29%)	44 (37%)	34 (28%)	143 (30%)
Grade 3	43 (36%)	52 (44%)	42 (36%)	54 (45%)	191 (40%)
Grade 4	44 (37%)	33 (28%)	32 (27%)	32 (27%)	141 (30%)
VAS - mean baseline	58.1	53.8	54.3	59.9	
95% CI	54.8-61.5	50.6-56.9	50.6-58.1	56.5-63.2	
KOOS pain - mean baseline	53.1	55.2	55.2	50.5	
95% CI	50.3–55.9	52.7–57.7	53.0–57.4	47.7–53.2	
Male, *n* (%)	56 (48%)	56 (47%)	53 (45%)	49 (41%)	214 (45%)
Female, *n* (%)	62 (53%)	63 (53%)	65 (55%)	71 (59%)	261 (55%)

### Primary outcome

**Fig. 2:**
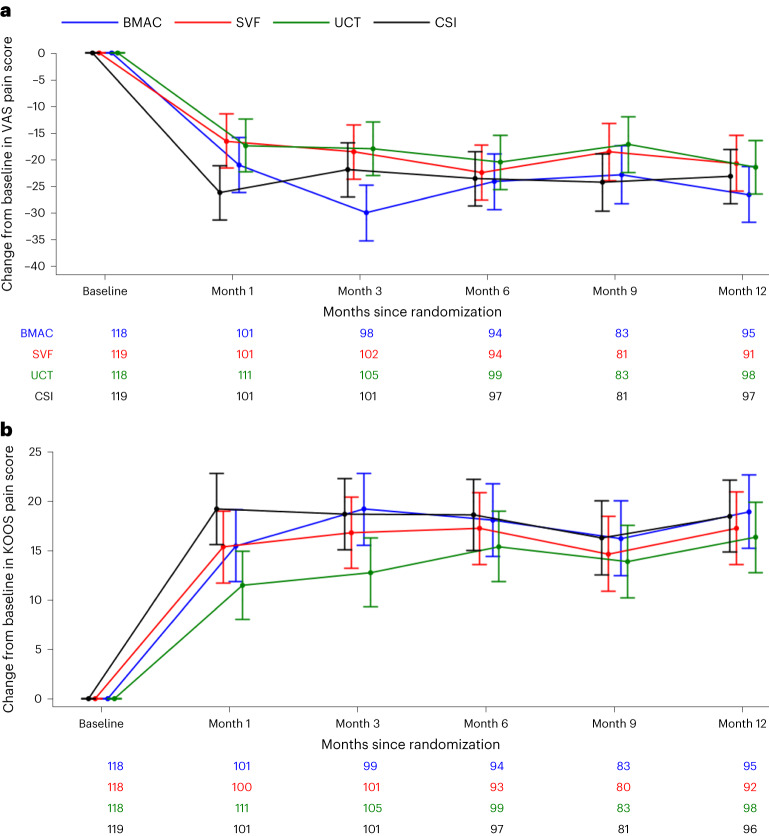
Primary outcome measures over time. **a**,**b**, Results of primary outcome change from baseline for VAS pain (**a**) and change from baseline for KOOS pain score (**b**) by treatment group and months since randomization. The time trend lines are the model-based means and 95% CIs. The vertical lines are the 95% CIs. Sample sizes for each treatment group at each time point are reported below each figure.

**Fig. 3:**
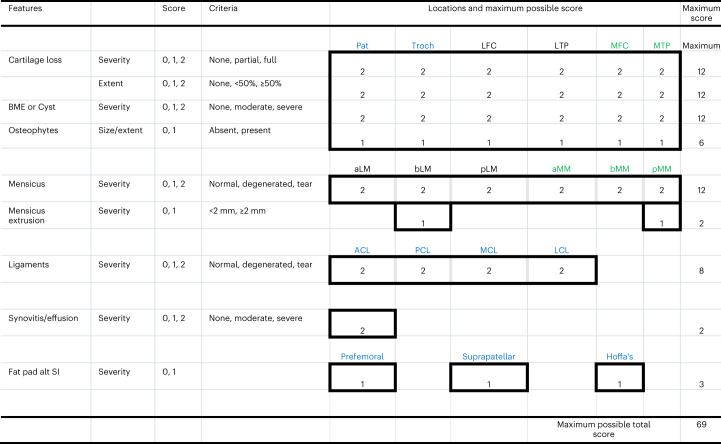
MRI scoring system used. The MRI is graded from 0 to 69, with the higher number representing more severe grades of OA. Features: BME (bone marrow edema) or Cyst, fat pad alt SI, fat pad signal intensity alteration. Location: Pat, patella; Troch, trochlea; LFC, lateral femoral condyle; LTP, lateral tibial plateau; MFC, medial femoral condyle; MTP, medial tibial plateau; aLM, anterior horn of lateral meniscus; bLM, body of lateral meniscus; pLM, posterior horn of lateral meniscus; aMM, anterior horn of medial meniscus; bMM, body of Medial Meniscus, pMM, posterior horn of medial meniscus; ACL, anterior cruciate ligament; PCL, posterior cruciate ligament; MCL, medial collateral ligament; LCL, lateral collateral ligament. Prefemoral, pre-femoral fat pad; Suprapatellar, suprapatellar fat pad; Hoffa’s, Hoffa’s fat pad. There was a total of 40 separate scores, adding up to the highest possible score of 69, representing the wort possible grade (that is, worst knee health). The MRI is graded from 0 to 69, with the higher number representing more severe grades of OA.

Both primary outcome measures results are shown in Fig. 2. The co-primary-outcome measure of VAS pain score was analyzed as the change from baseline to 12 months. The mean decline from baseline in VAS pain score changed in different ways by treatment group but was not significantly different in overall decline (for example, overall magnitude was same, but trajectory was different). There were no significant differences between groups at month 12 in the change in VAS score from baseline (change, −24.3 ± standard error of the mean (s.e.m.) in BMAC, −19.4 ± s.e.m. in SVF, −20.1 ± s.e.m. in UCT and −20.9 ± s.e.m. in CSI; difference versus CSI, BMAC: −3.4; *P* = 0.19, SVF: 1.5, *P* = 0.56, UCT: 0.8, *P* = 0.76). The analysis of KOOS pain score yielded similar results as there was no significant between-group difference at month 12 in the change in score from baseline (change, 19.1 ± s.e.m. in BMAC, 17.2 ± s.e.m. in SVF, 16.2 ± s.e.m. in UCT and 17.7 ± s.e.m. in CSI; difference versus CSI, BMAC: 1.4; *P* = 0.49, SVF: −0.50, *P* = 0.82, UCT: −1.5, *P* = 0.44). Prima sensitivity analyses were performed using the observed case, the per protocol population and multiple imputation under the assumption of missing not at random. For both the VAS pain score and the KOOS pain score, there was no significant between-group difference at month 12 in the change in score from baseline for any of the sensitivity analyses. Interaction between treatment group and age group (<60 versus ≥60), sex, ethnicity and Kellgren–Lawrence (KL) grade were also analyzed. There was a significant interaction between treatment group and age group *(P* = 0.02) and between treatment group and sex (*P* = 0.01) for VAS pain score. There was no significant interaction for KOOS pain score. In addition, the magnetic resonance imaging (MRI) scores had no significant changes from baseline in any of the four groups (BMAC 0.53, SVF −0.40, UCT −0.26 and CSI 0.30) compared to their baseline scores or compared to the CSI group (BMAC 0.23, SVF −0.69 and UCT −0.55). See Fig. 3 for description of MRI scoring system.

### Secondary outcome

In addition to our primary outcome measures we also analyzed EQ-5D and PROMIS-29 between cohorts and CSI. For EQ-5D, the treatment by time interaction was not significant (*P* = 0.26), suggesting EQ-5D in the four treatment groups changed in similar ways (similar temporal patterns for the four treatment groups). Since the interaction between treatment and time is not significant, we would not expect to see specific ‘change versus change’ differences. Similarly for PROMIS-29, we assessed all domains by a treatment by time interaction and there was no significance for the following subdomains: PROMIS-29 Anxiety (*P* = 0.78), Depression (*P* = 0.06), Fatigue (*P* = 0.56), Pain (*P* = 0.39), Physical Function (*P* = 0.048), Sleep (*P* = 0.91) and Social Rules and Activities (*P* = 0.82). It should be noted that the PROMIS-29 Physical Function was statistically significant, suggesting the physical function PROMIS-29 domain T-score in the four treatment groups changed in different ways (different temporal patterns for the four treatment groups). However no clear clinically important difference in the temporal pattern was apparent between the four treatment groups. The full details for these secondary outcomes are located in Supplementary Material 1.

### Exploratory outcomes

Bedside testing of total nucleated cells injected and viability for each cellular group was analyzed. In addition, 71 BMAC, 16 SVF and 8 UCT-MSC samples at the time of publication were subjected to single-cell RNA sequencing to reveal differences and similarities in the cellular components of each product via cell clustering analyses visualized in two dimensions using Uniform Manifold Approximation and Projection. Transcriptomic analyses at the single-cell resolution revealed that both autologous cell sources exhibited distinct cell subpopulations with some similarities in a subset of hematopoietic lineage-derived cells. Conversely, the UCT mesenchymal population, although exhibiting a defined clustering pattern, showed a uniform MSC phenotype.

### Safety

There were no procedure-related serious adverse events (AEs) reported, which includes any allergic reactions or symptomatic infections seen in any treated patient. However, there were multiple related AEs reported that have been subdivided. The following related AEs demonstrated significance between cohorts: joint swelling (CSI 7.4% versus UCT 24.1%, *P* = 0.01), post-procedural contusion (SVF 38.6% versus BMAC 12.2% versus UCT/CSI 0%, *P* < 0.0001), post-procedural hematoma (BMAC 2.9% versus SVF 12.4%, *P* = 0.02). For a list of the most common AEs by study arm and by study group, see Tables 2 and 3.

**Table 2:** Summary of AEs by treatment group according to MedDRA system

MedDRA system organ class (preferred term)	BMAC (*N* = 107)		SVF (*N* = 109)		UCT (*N* = 116)		CSI (*N* = 108)		All subjects (*N* = 440)	
	*n* (%)	95% CI	*n* (%)	95% CI	*n* (%)	95% CI	*n* (%)	95% CI	*n* (%)	95% CI
Musculoskeletal connective tissue disorders										
Arthralgia	27 (25.2)	17.3, 34.6	25 (22.9)	15.4, 32.0	30 (25.9)	18.2, 34.8	28 (25.9)	18.0, 35.2	110 (25.0)	21.0, 29.3
Joint stiffness	13 (12.1)	6.6, 19.9	6 (5.5)	2.0, 11.6	8 (6.9)	3.0, 13.1	8 (7.4)	3.3, 14.1	35 (8.0)	5.6, 10.9
Joint swelling	19 (17.8)	11.0, 26.3	16 (14.7)	8.6, 22.7	28 (24.1)	16.7, 33.0	8 (7.4)	3.3, 14.1	71 (16.1)	12.8, 19.9

The safety population is defined as the 440 subjects who received study treatment. The AEs are those with an incidence of at least 5%. MedDRA denotes Medical Dictionary for Regulatory Activities, version 21.1.

95% CIs that do not overlap are statistically significant.

**Table 3:** Summary of procedure related AEs by study arm

MedDRA system organ class (preferred term)	Arm 1 (BMAC bone marrow harvest) (*N* = 139)		Arm 2 (SVF fat harvest) (*N* = 145)		Arm 3 (UCT CSI no harvest) (*N* = 156)	
	*n* (%)	95% CI	*n* (%)	95% CI	*n* (%)	95% CI
Injury, poisoning and procedural complications						
Post-procedural contusion	17 (12.2)	7.3, 18.9	56 (38.6)	30.7, 47.1	0	0.0, 2.3
Post-procedural hematoma	4 (2.9)	0.8, 7.2	18 (12.4)	7.5, 18.9	0	0.0, 2.3
Procedural pain	41 (29.5)	22.1, 37.8	49 (33.8)	26.2, 42.1	1 (0.6)	0.0, 3.5

The safety population is defined as the 440 subjects who received study treatment. The AEs are those with an incidence of at least 5%. MedDRA denotes Medical Dictionary for Regulatory Activities, version 21.1.

95% CIs that do not overlap are statistically significant.

## Discussion

This study demonstrated no superiority of any cell therapy over corticosteroids at 1 year when VAS and KOOS were compared. In addition, other measures including EQ-5D and PROMIS also showed no superiority among cellular therapies over CSI. As such, the primary hypothesis was rejected. Given the complexity of the study, patients and cells involved, no direct knowledge was attained from our primary analysis about the personalization of cellular injections for patients.

The question of the most beneficial cellular treatment, and the assumption of superiority over CSI, has been debated for some time. However, large randomized clinical trials have been difficult to perform. Previously, the discussion over which source of cells are superior has been predominantly based on laboratory data analyzing MSCs, colony-forming units or other secretory factors. This type of approach is complicated by the large amount of heterogeneity among autologous and allogeneic products based on the donors who supply these cells. Based on in vitro analysis, the consensus is that birth tissue products will produce the higher number of true MSCs, but questions over the manufacturing, processing and efficacy of such treatments have been raised as most of the commercial products that have been available for use show no live MSCs^15,16^. However, autologous products have been shown to be extremely safe as same-day procedures and have consistently high cell viability. Our study has corroborated these findings and showed safety and tolerability of all the cellular therapies used. These cellular injection preparations, however, do not have large numbers of MSCs, and the therapeutic mechanism of action and overall efficacy have been called into question. Human clinical research has not clearly demonstrated success of these treatments, even with the most rigorous randomized control trial, which demonstrated equal improvement in pain with bilateral knee OA with BMAC injected to one knee and a saline injection in the other knee^9^. There have been, however, an abundance of case reports and case series in the literature that have shown favorable success and several meta-analyses that support the use of these products^17,18^.

A benefit of this large study was the evaluation of the safety of these procedures. Since this was a US Food and Drug Administration (FDA)-approved study, every adverse reaction, from pain and swelling in the joint to hospitalizations for illnesses unrelated to the study intervention, was recorded in real time. There were no study-related serious AEs or symptomatic knee infections noted in any of the treatment groups at any point during follow-up. The study-related AEs of procedural and post-procedural pain were highest in the SVF cohort followed by BMAC, and there were very few related AEs, none of which was severe. Almost all of the reported AEs recovered by the 1-week follow-up visit. This is consistent with previous reports of two large case series: one by Centeno et al in 2016 (ref. ^19^), the other by Hernigou in 2013 (ref. ^20^). These studies both examined BMAC procedures and AEs, and found, like us, that procedural pain or injection site pain were the only significant findings. Our study confirms an exceptional safety profile of these percutaneous procedures.

As none of the treatment groups was superior to another, it was deemed important to subgroup patients on the basis of their grade of arthritis. The use of KL scoring preinjection was used to classify patients accordingly. Despite having three types, II, III and IV, the KL grade was not a reliable predictor of success. The literature often points to a direct correlation between KL grading and specific product efficacy in small cohorts^21,22^. We speculate that the lack of differences among the KL grades may be due to the relative function of those with worse KL grades at the beginning of the trial or the large heterogeneity in age and sex that we recruited on the basis of our wide inclusion criteria aimed at decreasing bias. In no treatment group did we see any notable improvement in MRI scores when comparing corticosteroids to cellular injections. Moreover, the steroid group’s MRI scores did not significantly worsen over the 1-year mark and the cellular groups did not improve over that period. There were, however, certain MRIs that showed improvement over the 1-year period as evidenced by their MRI scoring. These changes will be further investigated in subsequent papers and with our extension study that will further characterize MRI changes 2 and 3 years post treatment. It should be noted that OA is a slow progressive process, and it is possible that a 1-year timeline was too short for this secondary measure.

Significant measures were taken to eliminate potential bias in our study, including blinding of authors to original data before final analysis. The primary analyses and the sensitivity analyses allowed us to evaluate all scenarios including subject loss, as we designed the study as intent to treat. However, selection bias may arise from differences between participants who selected to start the trial and those who did not. With interventions for knee OA, there is a well-described placebo effect that we must acknowledge. When trying to account for this placebo effect, one must look at studies where saline was used. It is debatable as to whether saline is a true control or has some therapeutic effect by diluting and washing away inflammatory mediators as well as aspiration of joint fluid, which occurs before any intervention. It is unclear whether different solutions and cellular therapies have this same dilution effect or if there is a reactionary byproduct from cellular injectates over saline. This placebo effect has been studied extensively in this area, with overall data suggesting there could be upwards of 50% response rate as well as up to 6 months improvement in pain and possibly even longer improvement in function^23,24^. It is unclear if there was a placebo effect given the fact that patients were blinded to their treatment and underwent sham procedures (for example, they may have assumed it worked because they got ‘stem cells’ or did not work because they got ‘steroids’). There were clearly some patients who did not respond to cellular or steroid therapies, and it is unclear whether these patients were subject to a placebo type effect^25^.

Although no study is perfect, our study identified that at 1 year post injection there was no cellular therapy that was more effective than CSI for knee OA. In addition, the study demonstrates in a large cohort the relative safety of these cellular procedures without evidence of severe AEs. It should be noted that a multiarm clinical trial evaluating the safety and efficacy of cellular biologics as compared to corticosteroid is exceedingly complex with multiple viewpoints and perceptions. A large team of scientists including expert opinion, critiques and requirements from the FDA shaped this study into its current form. Additional conclusions and responder analyses will be discussed more in future papers.

## Methods

### Study design and participants

This was a multicenter single-blinded study performed in accordance with guidelines and oversight from the FDA, and under the management of a contracted research organization. Study approval was obtained from the Western Institutional Review Board and by Duke and Emory University’s institutional review board. In accordance with the FDA, an investigational new drug application was filed, #18414, which referenced investigator device exemption #17894. The study was also registered with ClinicalTrials.gov, NCT03818737. Data availability at this time is available by request to the corresponding author(s).

A total of 570 patients were screened to identify 480 eligible patients who were randomized at five clinical sites in five different states within the United States. The five clinical sites included clinics from Emory, Sanford (two sites), Andrews Institute, and Duke. For a consort diagram of participants entered in study, see Fig. 1. Participants were randomized to a four-cohort parallel-design study. To allow for blinding, participatnts were further subdidivided to three arms given the need to ‘sham’ harvest cells from subjects in the SVF and BMAC arm, but not in the UCT/CSI arm: arm 1—autologous bone marrow concentrate (BMAC) (*n* = 120), corticosteroid (CSI) (*n* = 40); arm 2—SVF (*n* = 120), CSI (*n* = 40); arm 3—umbilical cord tissue (UCT) (*n* = 120), CSI (*n* = 40). The control cohort of CSI was then aggregated to allow a 1:1:1:1 comparision for analysis. Subjects were enrolled if they were between 40 and 70 years of age and carried a diagnosis of knee OA as determined by radiographs within 3 months of their clinical visit. A full list of eiligibility criteria is provided on ClinicalTrials.gov, NCT03818737. A total of five samples did not meet release criteria, including one BMAC and four SVF. This was secondary to failure of endotoxin testing and not formal cell count. Additionally, the following failed to complete the study. In the BMAC cohort, a total of 12 subjects did not complete the stud, with 1 related to release criteria as above, 10 related to subject withdrawal and 1 lost to follow-up. In the SVF cohort, a total of 12 subjects did not complete the study, with 1 due to investigator withdrawal, 4 related to release criteria as above, 6 related to subject withdrawal and 1 due to screen fail. In the UCT cohort, a total of 13 subjects did not complete the study, with 2 due to investigator withdrawal, 10 related to subject withdrawal and 1 lost to follow-up. Lastly, in the CSI cohort, a total of 11 subjects did not complete the study, with 1 due to investigator decision, 6 related to subject withdrawal and 4 lost to follow-up. Patients returned to clinic for MRI to assess cartilage and joint health at baseline, 6 months and 1 year. They were compensated US$50 for each MRI that was performed.

### Randomization and masking

Subjects were stratified by clinical center, and after obtaining informed consent and verifying eligibility criteria were met, subjects were randomized. Treatment assignments were stratified by clinical center and generated using a pseudo-random number generator with randomly permutated blocks. These assignments were stored in the Medidata Rave cloud-based data management system developed by the contracted research organization. All subjects underwent the harvesting procedure per their assigned study arm, then per the randomization scheme they were blinded to the actual treatment received (for example, SVF versus CSI, BMAC versus CSI, or cells versus CSI). As a single-blinded study, the site principal investigators were not required to be blinded. However, subjects were blinded to their injection. The blinding was implemented by limiting visualization of the syringe contents with opaque covering in addition to performing sham BMAC and SVF harvests in patients within certain cohorts.

### Procedures

The cellular harvesting, final product preparation, injection procedures and CSI were standardized across the five study sites. This was done through training courses before study initiation as well as subsequent monitoring by the lead site. Of note, the BMAC and SVF were fresh autologous products while the UCT were cryopreserved, purified MSCs manufactured from donated allogeneic umbilical cord tissue in a cGMP (current Good Manufacturing Practice) facility. Supplementary Material 1 details the contents of each preparation that was injected.

All procedures were done in an outpatient clinic setting with another clean room used for point-of-care laboratory testing. No cGMP facility was used for the actual treatment portion of this study. The subject was positioned supine on an examination table with foam roller/bolster under knee. The injectate was prepared by the research team with opaque tape wrapped around the 10-ml syringe to maintain the blinding. If an effusion was present based on clinical and ultrasound examination, 2 ml of ropivicaine 0.2% was injected between the skin and down to the joint capsule. Following this, an 18-gauge needle was used to aspirate any joint fluid via a superior lateral approach under direct ultrasound guidance. Following joint aspiration (if performed), the solution of either corticosteroids, BMAC, SVF or UCT was injected under ultrasound guidance. Following the injection, the knee was passively moved from extension to flexion three to four times to help spread the injectate and patients were instructed to remain supine for 10 min.

All final cellular products were tested in the clinic for total nucleated cell count, cell viability and endotoxin levels to determine if release criteria were met before injection. These tests were performed using a Nexcelom Auto 2000 device for cell counting and cell viability and the Charles River Endosafe Nexgen PTS for endotoxin testing. A 1-ml aliquot of each cellular product was separated from the final injection preparation for the release criteria testing and FDA requirements. If the final product did not meet the required release criteria depicted in Table 1, the subject did not receive the injection. This was seen in one BMAC patient and four SVF patients. In addition to bedside testing, 14-day sterility testing was performed post administration per FDA requirements. In an effort to standardize the injectate and per FDA guidance, cutoffs and cellular numbers were derived from a group of subject matter experts.

Cellular diversity in BMAC and SVF samples and cellular heterogeneity in UCT-MSC samples were evaluated by single-cell RNA sequencing. The single-cell RNA sequencing libraries were prepared with 10x Genomics Chromium platform using 3′ V3.1 kit. The sequencing was done on Illumina Novaseq 6000 platform with an S4 kit. We used a modified SEURAT pipeline to analyze the samples^26^. We then applied filters to include cells that had more than 800 unique molecular identifiers, more than 500 expressed genes and mitochondria percentage less than 20%.

### Outcomes

The primary analyses of the data were performed according to subjects’ original treatment assignment (that is, intention-to-treat analyses) regardless of their compliance and the inclusion of all data from all subjects randomized in the final analysis. The two co-primary efficacy endpoints in this intent-to-treat, parallel-group trial were VAS and KOOS pain score at 12-month visit from baseline.

KOOS is a self-reported outcome measure assessing the patient’s opinion about the health, symptoms and functionality of their knee. It is a 42-item questionnaire, including five subscales: symptoms, pain, activities of daily living, sports/recreation and quality of life. The maximum score a patient can achieve is 100, and the minimum score is zero, indicating severe knee problems. KOOS has been verified in assessing patients of various age populations, ranging from young to elderly adults^27^.

The VAS is a single-item measure that most commonly consists of a 100-mm horizontal line anchored with two opposite labels; patients mark a score on the scale using a vertical line^28^. Magnetic resonance images were evaluated by a musculoskeletal radiologist according to a semi-quantitative method for whole-knee analysis in the setting of OA. Our methodology, adopted from past work (that is, WORMS (1) and BLOKS (2) grading schemes) evaluated severity and/or extent of cartilage loss, bone marrow edema (BME) or cyst, osteophytes, meniscus pathology and extrusion, ligament pathology, synovitis, and fat pad inflammation^29,30^. Both WORMS and BLOKS were utilized for this study. T2 mapping is a technique to determine intrinsic spin-lattice relaxation times of biological tissues and has been studied extensively in the knee to assess cartilage and meniscus degeneration. For this project, we utilized spin echo T2 mapping technique, where four to eight images with echo times ranging from ~10 ms to ~80 ms were obtained in weight-bearing regions of the medial and lateral compartments. For the magnetic resonance scoring system that was used, see Fig. 3.

### Statistical analysis

Power and sample size considerations for the trial are found in Supplementary Material 1. For primary endpoint analyses, missing data for VAS pain scores and KOOS pain scores were imputed using multiple imputation under the missing at random assumption). Independent imputations were performed for VAS pain scores and KOOS pain scores. Absolute change from baseline in VAS pain score and KOOS pain score were derived from the corresponding imputed scores. Sensitivity analyses for the primary endpoints were performed using the observed case, the per protocol population and multiple imputation based on a pattern-mixture model under the assumption of missing not at random (all found in Supplementary Material 1.).

A repeated-measures analysis of change of VAS pain score (and change of KOOS pain score) was performed with a means model via the SAS MIXED Procedure (version 9.4; SAS Institute, Cary, NC), providing separate estimates of the means by treatment group (BMAC, SVF, UCT and CSI) time on study (1, 3, 6, 9 and 12 months on study) and treatment group. The models included treatment arm, time on study, the statistical interaction between treatment arm and time on study and study center as fixed effects. A compound-symmetric variance–covariance form in repeated measurements was assumed, and robust estimates of the standard errors of parameters were used to perform statistical tests and construct 95% confidence intervals (CIs)^31^. The model-based means are unbiased with unbalanced and missing data, if the missing data are non-informative (missing at random). The main effect test for treatment at the 12-month visit was used as the primary hypothesis test to compare the treatment arms. The primary study results from this model were the mean change score and 95% CI for each of the four treatment cohorts and the treatment mean differences and 95% CIs. Pairwise treatment comparisons on efficacy score change were performed. The primary comparisons were each cellular treatment against control group. A Hochberg adjustment method was used to maintain the overall *α* level, which ordered the *P* value from high to low and compared the largest *P* value to 0.05, the middle *P* value to 0.05/2 and the smallest *P* value to 0.05/3 (ref. ^32^). Specific statistical tests were done within the framework of the mixed effects linear model. All statistical tests were two-sided. Secondary efficacy endpoint analyses (all VAS, KOOS and EQ-5D 3L scores) were conducted using an observed case analysis using the same plan described for the change scores. No interim analyses for efficacy were performed for this study.

The heterogeneity of treatment effects across levels of a baseline variable was investigated using a statistical test for interaction. A prespecified subgroup analysis is one that is planned and documented before examination of the data. Planned subgroup analyses were performed to examine the impact of treatment on the primary outcomes in the prespecified subgroups (that is, sex, ethnicity and KL grade). Effect of treatment in subgroups was determined by including the interaction between treatment and subgroup in the repeated-measures model described above using an observed case analysis. Additional secondary and exploratory outcome measures included change in MRI cartilage and joint health from baseline to 1 year, analysis of AEs and complications between groups, as well as in-depth cellular analysis of each injectate.

### Reporting summary

Further information on research design is available in the Nature Portfolio Reporting Summary linked to this article.

## Online content

Any methods, additional references, Nature Portfolio reporting summaries, source data, extended data, supplementary information, acknowledgements, peer review information; details of author contributions and competing interests; and statements of data and code availability are available at 10.1038/s41591-023-02632-w.

### Supplementary information

Supplementary Information Supplementary Material 1–4 (injection preparations, supplementary methods, additional results and study protocol).

Reporting Summary

## Data Availability

Upon discussion with our study leadership, we plan to make data available by request only initially, and will make publicly available following completion of additional manuscripts that are still to be submitted. In addition, significant portions of our data are available in our Supplementary Information. Submit data requests to corresponding authors K.M. and H.D.
