# Author Correction: Cell-based versus corticosteroid injections for knee pain in osteoarthritis: a randomized phase 3 trial

**DOI:** 10.1038/s41591-023-02776-9

**Published:** 2023-12-22

**Authors:** Ken Mautner, Michael Gottschalk, Scott D. Boden, Alison Akard, Won C. Bae, Lora Black, Blake Boggess, Paramita Chatterjee, Christine B. Chung, Kirk A. Easley, Greg Gibson, Josh Hackel, Katie Jensen, Linda Kippner, Chad Kurtenbach, Joanne Kurtzberg, R. Amadeus Mason, Benjamin Noonan, Krishnendu Roy, Verle Valentine, Carolyn Yeago, Hicham Drissi

**Affiliations:** 1 Department of Orthopaedics, Emory University, Atlanta, GA USA; 2 Department of Radiology, University of California, Davis, La Jolla, CA USA; 3 Sanford Health, Sioux Falls, SD USA; 4 Duke Sports Medicine, Durham, NC USA; 5 Marcus Center for Therapeutic Cell Characterization and Manufacturing, Georgia Institute of Technology, Atlanta, GA USA; 6 Department of Biostatistics and Bioinformatics, Emory University, Atlanta, GA USA; 7 Center for Integrative Genomics, Georgia Institute of Technology, Atlanta, GA USA; 8 Andrews Institute, Gulf Breeze, AL USA; 9 Marcus Center for Therapeutic Cures, Duke University, Durham, NC USA; 10 Sanford Health, Fargo, ND USA; 11 Biomedical Engineering, Vanderbilt University, Nashville, TN USA

**Keywords:** Mesenchymal stem cells, Stem-cell research

Correction to: *Nature Medicine* 10.1038/s41591-023-02632-w, published online 2 November 2023.

In the version of the article initially published, Table 1 was missing two rows (“VAS – mean baseline” and “KOOS pain – mean baseline”) that have now been inserted in the HTML and PDF versions of the article.

