# Supplementary material for: Cell-based versus corticosteroid injections for knee pain in osteoarthritis: a randomized phase 3 trial
Source: Nat Med. 2023 Nov 2;29(12):3120–6. doi: 10.1038/s41591-023-02632-w (PMC10719084; doi:10.1038/s41591-023-02632-w)
Supplement: Supplementary file 1 — Supplementary Material 1–4 (injection preparations, supplementary methods, additional results and study protocol). [file 41591_2023_2632_MOESM1_ESM.pdf]

nature medicine

Article
https://doi.org/10.1038/s41591-023-02632-w

Cell-based versus corticosteroid injections for knee pain in osteoarthritis: a randomized phase 3 trial

In the format provided by the authors and unedited

Details of the contents of each injection group

Corticosteroid

A 10mL syringe containing 1mL of depomedrol (40mg/dL) and 6mL of normal saline was prepared.

Bone Marrow Concentrate (BMC)

BMC was prepared by harvesting of bone marrow aspirate (BMA) from the posterior superior iliac spine (PSIS). A total of 61mL of bone marrow was procured under ultrasound- guidance from bilateral PSIS via 3 passes on each side, at least 1 cm apart, through the bone cortex. Approximately 10.33mL was obtained from each of the 6 sites. Following this, centrifugation occurred in an FDA approved device (EmCyte GenesisCS Pure BMAC®-60 mL). The aspirate was separated into a single concentrating device, counterbalanced, and placed in a centrifuge for 2.5 minutes at 3800 RPM. The plasma and cell concentrate were separated with a syringe and stopcock, transferred into the concentrating accessory, counterbalanced, and re-spun for 7 minutes at 3800 RPM to separate the cell concentrate from the plasma. After the plasma was drawn off and swirled to re-suspend, the BMC was drawn into a syringe for injection into the patient's knee joint. The BMC production procedure results in 8 mL of product with 1mL removed and sent for characterization (along with 1cc of BMA) and 7 mL prepared for final injection.

Stromal Vascular Fraction (SVF)

Stromal Vascular Fraction (SVF) was prepared at the point of care using the enzymatic Transpose RT / Matrase system (InGeneron, Houston, TX, USA). Following sterile preparation, a tumescent solution of lidocaine with epinephrine and normal saline was injected into the subcutaneous fat tissue where lipoaspiration occurred. After waiting 15-20 minutes for proper analgesia and vasoconstriction, a minimum of 100 mL was aspirated via a syringe lipoplasty procedure. The excess tumescent solution was allowed to separate from the adipose tissue and discarded prior to isolation of the SVF. For the isolation of the SVF, lipoaspirate was combined with Matrase Reagent (a specific, proprietary, GMP produced enzyme blend of collagenases and neutral protease; InGeneron) and lactated ringers- or saline solution in sterile single-use Transpose Processing Tubes. The processing tubes were placed in the Transpose Tissue Processing Unit and processed for 30 minutes through repetitive processing cycles at 38° Celsius. This initial step dissolved the extracellular matrix and released the individual cells of the SVF from their niche. The cellular suspension was extracted with a 60 mL syringe and transferred to the Transpose Wash Tubes through a cell strainer to remove non cellular components and residual extracellular matrix. The Wash Tubes were centrifuged in the processing unit to concentrate the cells of the SVF at the bottom valve. This process separated the contents of the processed adipose tissue into oils and lipids released from adipocytes, extracellular matrix and remaining adipocytes, the aqueous phase containing remaining tumescent solution, saline and residual Matrase and the cells of the SVF. The SVF cells were extracted through the valve into a 3mL syringe, and the supernatant containing the Matrase,

extracellular matrix, adipocytes and lipids was discarded. The cells of the SVF were returned to the wash tube through the luer connected syringe and suspended in saline solution. The cells were then centrifuged for another 5 minutes, and this step was repeated once (these two steps are designed to remove the residual enzyme and remaining collagen particles). After completion of the processing, in a 10 mL Luer Lock syringe, 6 mL containing the cells of the SVF were collected from the wash tubes. The resulting suspended cell mixture was split into 1 mL for release testing and the remaining 5 mL of the SVF were prepared for injection into the subjects.

Umbilical Cord Tissue (UCT)

hCT-MSCs are a product of allogeneic cells manufactured from digested umbilical cord tissue that is expanded in culture, cryopreserved and banked. hCT-MSCs are manufactured from umbilical cord tissue donated to the Carolinas Cord Blood Bank, an FDA-licensed, FACT-accredited, public cord blood bank at Duke University Medical Center (BLA 125407 DUCORD), after written informed consent from the baby's mother. Cord tissue is harvested from the placentas of male babies delivered by elective C-section after a normal, full-term pregnancy at the Duke Medical Center collection sites affiliated with the Carolinas Cord Blood Bank. Donor screening questionnaires are completed by the maternal donor, and maternal blood is tested for communicable diseases by the CLIA-certified donor screening laboratory per 21 CFR Part 1271. Donors must be eligible for donation to a public cord blood bank for allogeneic use. In the operating room, after aseptic delivery of the placenta and cord, the cord blood is aseptically drained from the placenta. Then the cord is dried and cleaned with chloropreps (Henry Schein, Melville, NY), separated from the base of the placenta, placed in a sterile bottle containing Plasmalyte A (Baxter Healthcare,Deerfield, IL), and transported to the Robertson GMP cell processing laboratory at room temperature in a validated container for additional processing on the same day.

In the clean room manufacturing suite, in a biosafety cabinet, the cord tissue is removed from the media, placed in sterile dishes, cut into small pieces, and then minced and digested in the Miltenyi Biotec GentleMacs Octo Dissociator with 4 GMP-grade enzymes (proprietary). The resultant cell suspension is placed in culture in Prime XV MSC Expansion XSFM (Fujifilm,Irvine Scientific, Santa Ana, CA) media with 1% platelet lysate (Compass Biomedical, Hopkinton, MA) and grown to confluence (~7-14 days) to establish the P0 culture. To establish the master cell bank, P0 is harvested and cryopreserved in cryovials with Cryostor 10 media (BioLife, Bothell, WA), and stored in the vapor phase of liquid nitrogen. P1 and P2 cultures are grown under similar conditions over 14-16 days, in hyperflasks without platelet lysate, as needed to create the working cell bank and product for administration, respectively. Cells from P1 and P2 are removed from plastic cultureware using 10% TrypLE (Gibco, Billings, MT). The final product is derived from the P2 cultures which are harvested into plasmalyte A with 5% human serum albumin, washed and cryopreserved in 5 compartment cryobags (Pall Corporation, Port Washington, New York) containing 25-50 million cells in 5 mL at a final concentration of 10% DMSO in 50% $dextran_{40}$ (Akron Biotechnology, Boca Raton, FL). These cells are frozen in a controlled rate freezer and stored under liquid nitrogen until shipment to the treatment center.

At each passage, the cell product is characterized by assessing cell surface phenotype by flow cytometry and functional assays via T-cell proliferation and organotypic models of microglial activation. Each lot, prior to cryopreservation of P2, was tested for sterility, endotoxin and mycoplasma and these tests must meet specifications for dosing, release testing after thaw and dilution includes TNCC and viability via cellometer. Cells were transported in a dry shipper and thawed at the point of care. After thawing, 2-2.5mL of solution remained and is aspirated from the cryobag, suspended in saline to produce 7mL which is then prepared for injection into the subject.

Release Criteria

All final cellular products were tested in the clinic for total nucleated cell count, cell viability, and endotoxin levels to determine if release criteria were met prior to injection. These tests were performed using a Nexcelom Auto 2000 device for cell counting and cell viability and the Charles River Endosafe Nexgen PTS for endotoxin testing. A 1mL aliquot of each cellular product was separated from the final injection preparation for the release criteria testing and FDA requirements. If the final product did not meet the required release criteria as depicted below the subject did not receive the injection.

**Release Criteria**	**SVF**	**BMAC**	**UCT**
Total Nucleated Cell Count	>5 million cells (no maximum cell count)	>200 million cells but <2 billion cells	>10 million cells but < 20 million cells
Cell Viability	≥70%	≥70%	≥70%
Endotoxin Testing	<20 EU total	< 350 EU total	Not required at point of care

Supplemental Methods

Sample Size and Power Considerations

The total estimated sample size for the proposed intention-to treat, parallel-group, multicenter, randomized, controlled trial is 480 subjects. The primary endpoints are Visual Analog Scale (VAS) pain score and the pain subsection of Knee Injury and Osteoarthritis Outcomes (KOOS). The sample size calculations are based on improvements (from baseline to 1-year) in VAS pain score and total KOOS. KOOS total score was used as a proxy and should ensure the study is amply powered.

Considering a 10-point scale, assuming a decline of 1 point on average in pain in the Control arm (corticosteroids) and a decline on average of 2.5 points in a treatment arm (mesenchymal stem cells) and an estimated standard deviation on change of 3.5, the proposed sample sizes (n=121 subjects per group or 484 total subjects) will provide 91% power to detect a difference on change of 1.5 points at the two-sided 5% significance level if the true difference between treatment arms is 1.5 points (two-sided two-sample equal variance t-test).

Assuming an increase of 10 points on average in total KOOS in the Control arm (corticosteroids) and an increase on average of 20 points in a treatment arm (mesenchymal stem cells) and an estimated standard deviation on change of 20, the proposed sample sizes (n=121 subjects per group or 484 total subjects) will provide 97% power to detect a difference on change of 10 points at the two-sided 5% significance level if the true difference between treatment arms is 10 points (two-sided two-sample equal variance t-test).

Each of the four participating sites will accrue 30 subjects to each of the 4 treatment arms. Cohort retention is expected to be 90% at 1-year.

Imputation methods using the SAS MI procedure and the SAS MIANALYZE procedure [eREF1]:
Independent imputations were performed for VAS pain scores (over 1 Month) and KOOS pain scores. Absolute change from baseline in VAS pain score (over 1 Month) and KOOS pain score were derived from the corresponding imputed scores. For VAS pain scores (over 1 Month) and KOOS pain scores, the missingness pattern in the data was evaluated. When the pattern was not monotone, the Monte Carlo Markov Chain (MCMC) method of the SAS MI Procedure was used to make the pattern monotone. The SAS Procedure MI was used for imputing missing values of data with the monotone missing pattern. When the MCMC method was previously employed, one imputation was made using each of the fifty MCMC-imputed datasets. When the MCMC method was not previously employed, fifty imputations were created assuming the data was missing at random. These imputations used the following model: For VAS pain scores (over 1 Month) and KOOS pain scores, a linear regression model was used with covariates for treatment and non-missing VAS pain score (over 1 Month) and KOOS pain score from earlier scheduled time points including baseline. The resulting analysis on the imputed datasets was then combined to produce a single set of statistics by combining results from the mixed model for repeated measures analysis using the SAS MIANALYZE Procedure.

Pattern-Mixture Model [eREF2]:
For VAS pain scores (over 1 Month) and KOOS pain scores, the missingness pattern in the data was evaluated. When missingness follows a pattern of dropout, pattern-mixture models can be used. These models work by conducting the analysis on a mixture of distributions from groups of patients based on their distribution of responses. Within each treatment group, the missing data are assumed to be ignorable. It is assumed that nonignorable missing data within a treatment group is a small proportion of the data in the group and that there is a negligible impact on the effect on the mean estimates within that treatment group. The following sensitivity analyses were performed when there was sufficient evidence to demonstrate that the missingness pattern differed across treatment groups. Analysis of absolute change in VAS pain score (over 1 Month) and KOOS pain score from baseline to month 12 was repeated on the ITT population by imputing missing data using multiple imputation based on a pattern-mixture model under the missing not at random (MNAR) assumption, using the profiles from corticosteroids subjects with observed data to impute missing data. The process was the same described for the missing at random analysis, only the assumption about missing mechanism was changed.

Generalized Estimating Equations for correlated binary data (responder analyses)
Responders were defined as all subjects who had an improvement in score (VAS or KOOS) of at least 25% or 50% and at least 20 points absolute change at a visit. Overall responders were defined as subjects who had a response for at least one study visit. For each endpoint and definition of responder, the generalized estimating equations (GEE) approach of Liang and Zeger [**eREF3**] was used to analyze the longitudinal data. A GEE regression analysis was performed with treatment, age group, gender, K/L grade, body mass index and treatment by visit interaction included as predictive variables. The analysis was implemented using the SAS procedure GENMOD with an exchangeable correlation structure for the repeated binary responses within patient (binomial distribution with the logit link function). Odds ratios and the associated confidence intervals and p-values by visit were obtained from the GEE regression model. Odds ratios were also obtained for overall responders. The same model was also analyzed by splitting the data by age group, gender, and K/L grade. K/L grades were dichotomized (2 vs 3/4, 3 vs 2/4 and 4 vs 2/3).

eReferences:

1. Little RJA, Rubin DB. *Statistical Analysis with Missing Data*. Hoboken: John Wiley & Sons; 2014.

2. Little RJA. Pattern-mixture models for multivariate incomplete data. *J Am Stat Assoc*. 1993;88(421):125-134.

3. Liang KY and Zeger SL. Longitudinal data analysis using generalized linear models. Biometrika 73:13-22, 1996.

Repeated measures analyses for secondary outcomes: Longitudinal change in EQ-5D and PROMIS T scores for each domain by treatment group and time on study.

		Time<br>Mean ± SEM<br>n (sample size)						P Values		
**Outcome**	**Treatment**	**Baseline**	**Month 1**	**Month 3**	**Month 6**	**Month 9**	**Month 12**	**Treatment by Time Interaction**	**Treatment**	**Time**
EQ-5D	BMAC	0.757 ± 0.013 n=118	0.834 ± 0.013 n=100	0.831 ± 0.013 n= 98	0.828 ± 0.014 n= 94	0.795 ± 0.014 n= 83	0.834 ± 0.014 n= 95	0.256	0.320	<.001
	SVF	0.778 ± 0.013 n=118	0.834 ± 0.013 n=101	0.833 ± 0.013 n=102	0.832 ± 0.014 n= 94	0.828 ± 0.014 n= 81	0.833 ± 0.014 n= 92	.	.	.
	UCT	0.752 ± 0.013 n=117	0.816 ± 0.013 n=111	0.818 ± 0.013 n=105	0.804 ± 0.013 n= 99	0.824 ± 0.014 n= 83	0.839 ± 0.013 n= 97	.	.	.
	CSI	0.746 ± 0.012 n=118	0.813 ± 0.013 n=102	0.827 ± 0.013 n=101	0.807 ± 0.013 n= 98	0.804 ± 0.014 n= 81	0.795 ± 0.013 n= 97	.	.	.

PROMIS T Scores		Time<br>Mean ± SEM<br>n (sample size)						P Values		
PROMIS domain	Treatment	Baseline	Month 1	Month 3	Month 6	Month 9	Month 12	Treatment by Time Interaction	Treatment	Time
PROMIS29 Anxiety T Score	BMAC	46.49 ± 0.74 n=117	45.47 ± 0.78 n=100	44.61 ± 0.78 n= 99	45.32 ± 0.79 n= 94	45.67 ± 0.82 n= 82	45.01 ± 0.79 n= 95	0.783	0.982	<.001
	SVF	47.17 ± 0.74 n=117	45.07 ± 0.77 n=101	45.54 ± 0.77 n=102	45.86 ± 0.78 n= 94	45.24 ± 0.81 n= 81	45.24 ± 0.79 n= 92	.	.	.
	UCT	46.94 ± 0.74 n=117	45.71 ± 0.75 n=111	45.41 ± 0.76 n=104	45.26 ± 0.77 n= 99	44.81 ± 0.81 n= 83	44.77 ± 0.77 n= 98	.	.	.
	CSI	48.08 ± 0.73 n=119	45.42 ± 0.77 n=101	45.20 ± 0.77 n=101	45.27 ± 0.78 n= 97	45.49 ± 0.81 n= 81	44.85 ± 0.78 n= 97	.	.	.
PROMIS29 Depression T Score	BMAC	44.31 ± 0.61 n=117	44.08 ± 0.64 n=100	43.64 ± 0.64 n= 99	43.62 ± 0.65 n= 94	45.29 ± 0.67 n= 82	44.28 ± 0.65 n= 95	0.060	0.864	0.069
	SVF	44.86 ± 0.61 n=117	43.81 ± 0.63 n=101	44.15 ± 0.63 n=102	44.71 ± 0.65 n= 94	44.92 ± 0.67 n= 81	43.92 ± 0.65 n= 92	.	.	.

PROMIS T Scores		Time Mean ± SEM n (sample size)						P Values		
**PROMIS domain**	**Treatment**	**Baseline**	**Month 1**	**Month 3**	**Month 6**	**Month 9**	**Month 12**	**Treatment by Time Interaction**	**Treatment**	**Time**
	UCT	44.83 ± 0.61 n=117	44.83 ± 0.62 n=111	44.42 ± 0.63 n=105	44.03 ± 0.64 n= 99	43.45 ± 0.66 n= 83	44.32 ± 0.64 n= 98	.	.	.
	CSI	45.64 ± 0.61 n=119	45.21 ± 0.63 n=102	44.70 ± 0.63 n=101	44.27 ± 0.64 n= 97	44.37 ± 0.67 n= 80	44.46 ± 0.64 n= 97	.	.	.
PROMIS29 Fatigue T Score	BMAC	46.65 ± 0.83 n=117	45.90 ± 0.87 n=100	45.10 ± 0.87 n= 99	46.00 ± 0.88 n= 93	46.86 ± 0.91 n= 82	45.76 ± 0.88 n= 95	0.564	0.878	<.001
	SVF	47.10 ± 0.83 n=116	45.84 ± 0.86 n=101	45.38 ± 0.86 n=102	46.29 ± 0.88 n= 93	46.52 ± 0.91 n= 81	45.31 ± 0.88 n= 92	.	.	.
	UCT	47.84 ± 0.83 n=117	47.11 ± 0.84 n=111	46.36 ± 0.85 n=105	45.51 ± 0.86 n= 99	47.41 ± 0.90 n= 83	46.22 ± 0.87 n= 98	.	.	.
	CSI	48.57 ± 0.82 n=119	46.54 ± 0.86 n=102	45.11 ± 0.86 n=101	45.44 ± 0.87 n= 97	47.04 ± 0.91 n= 80	45.39 ± 0.87 n= 97	.	.	.

PROMIS T Scores		Time<br>Mean ± SEM<br>n (sample size)						P Values		
**PROMIS domain**	**Treatment**	**Baseline**	**Month 1**	**Month 3**	**Month 6**	**Month 9**	**Month 12**	**Treatment by Time Interaction**	**Treatment**	**Time**
PROMIS29 Pain T Score	BMAC	58.54 ± 0.74 n=117	53.27 ± 0.78 n= 98	51.33 ± 0.78 n= 99	51.31 ± 0.79 n= 93	52.23 ± 0.82 n= 82	51.55 ± 0.79 n= 95	0.399	0.161	<.001
	SVF	58.17 ± 0.74 n=117	52.61 ± 0.77 n=101	52.19 ± 0.77 n=102	51.62 ± 0.79 n= 94	52.68 ± 0.82 n= 81	50.90 ± 0.79 n= 92	.	.	.
	UCT	58.56 ± 0.74 n=117	54.53 ± 0.75 n=111	52.49 ± 0.76 n=105	52.00 ± 0.77 n= 99	52.28 ± 0.81 n= 82	51.55 ± 0.77 n= 98	.	.	.
	CSI	60.08 ± 0.73 n=118	53.48 ± 0.77 n=101	53.66 ± 0.77 n=101	53.87 ± 0.78 n= 98	53.73 ± 0.82 n= 80	53.06 ± 0.78 n= 97	.	.	.
PROMIS29 Physical Function T Score	BMAC	39.72 ± 0.62 n=116	43.37 ± 0.65 n= 99	45.06 ± 0.65 n= 99	44.70 ± 0.66 n= 94	43.02 ± 0.68 n= 82	44.27 ± 0.65 n= 95	0.048	0.173	<.001
	SVF	40.68 ± 0.61 n=117	43.62 ± 0.64 n=100	44.23 ± 0.64 n=100	45.07 ± 0.65 n= 93	44.35 ± 0.68 n= 81	45.19 ± 0.66 n= 91	.	.	.
	UCT	39.05 ± 0.61 n=117	42.23 ± 0.62 n=111	43.89 ± 0.63 n=105	44.16 ± 0.64 n= 99	44.10 ± 0.67 n= 83	44.60 ± 0.64 n= 98	.	.	.

PROMIS T Scores		Time<br>Mean ± SEM<br>n (sample size)						P Values		
**PROMIS domain**	**Treatment**	**Baseline**	**Month 1**	**Month 3**	**Month 6**	**Month 9**	**Month 12**	**Treatment by Time Interaction**	**Treatment**	**Time**
	CSI	38.50 ± 0.61 n=119	43.30 ± 0.64 n=101	43.03 ± 0.64 n=102	43.02 ± 0.64 n= 97	42.71 ± 0.67 n= 81	43.43 ± 0.64 n= 97	.	.	.
PROMIS29 Sleep T Score	BMAC	49.09 ± 0.75 n=116	47.57 ± 0.78 n=100	47.76 ± 0.79 n= 99	48.86 ± 0.80 n= 93	49.24 ± 0.83 n= 82	48.36 ± 0.79 n= 95	0.913	0.894	<.001
	SVF	49.89 ± 0.75 n=116	48.46 ± 0.78 n=101	48.48 ± 0.77 n=102	48.83 ± 0.79 n= 94	48.61 ± 0.82 n= 81	48.92 ± 0.80 n= 92	.	.	.
	UCT	50.43 ± 0.75 n=117	48.55 ± 0.76 n=111	48.50 ± 0.77 n=104	48.53 ± 0.78 n= 99	49.01 ± 0.81 n= 83	49.19 ± 0.78 n= 98	.	.	.
	CSI	50.28 ± 0.74 n=119	49.26 ± 0.77 n=102	48.13 ± 0.77 n=102	49.01 ± 0.78 n= 98	48.78 ± 0.82 n= 80	48.99 ± 0.78 n= 97	.	.	.

**PROMIS T Scores**		**Time Mean ± SEM n (sample size)**						**P Values**		
**PROMIS domain**	**Treatment**	**Baseline**	**Month 1**	**Month 3**	**Month 6**	**Month 9**	**Month 12**	**Treatment by Time Interaction**	**Treatment**	**Time**
PROMIS29 Social Rules and Activities T Score	BMAC	49.82 ± 0.80 n=117	52.46 ± 0.84 n= 98	54.77 ± 0.84 n= 99	53.52 ± 0.86 n= 93	53.67 ± 0.89 n= 82	53.23 ± 0.85 n= 95	0.824	0.137	<.001
	SVF	48.18 ± 0.79 n=117	51.49 ± 0.83 n=101	53.17 ± 0.83 n=102	52.75 ± 0.85 n= 94	52.54 ± 0.88 n= 81	53.50 ± 0.85 n= 92	.	.	.
	UCT	48.29 ± 0.79 n=117	51.05 ± 0.81 n=109	52.35 ± 0.82 n=105	52.08 ± 0.83 n= 99	52.16 ± 0.88 n= 82	53.28 ± 0.84 n= 98	.	.	.
	CSI	47.11 ± 0.79 n=119	51.27 ± 0.83 n=101	51.52 ± 0.83 n=102	51.75 ± 0.83 n= 98	51.86 ± 0.88 n= 80	51.76 ± 0.84 n= 97	.	.	.

Specific statistical tests were done within the framework of the mixed effects linear model. All statistical tests were 2-sided. No adjustment for multiple comparisons was needed for the P values reported in this table (only one statistical test was performed for each of the three effects reported in the table for each secondary outcome).

The treatment by time interaction for EQ-5D was not significant (P = 0.26) suggesting EQ-5D in the four treatment groups changed in similar ways (similar temporal patterns for the four treatment groups). Since the interaction between treatment and time is not significant, we would not expect to see specific 'change versus change' differences between treatment groups. The time-averaged treatment means for EQ-5D were not different (P = 0.32).

Similarly, for PROMIS 29, we assessed all domains by a treatment by time interaction and there was no significance for the following subdomains: PROMIS-29 Anxiety (P=0.78), Depression (P=0.06), Fatigue (P=0.56), Pain (P=0.39), Physical Function (P=0.048), Sleep (P=0.91), and Social Rules and Activities (P=0.82). It should be noted that the PROMIS-29 Physical Function was statistically significant suggesting the physical function PROMIS-29 domain T-score in the four treatment groups changed in different ways (different temporal patterns for the four treatment groups). However no clear clinically important difference in the temporal pattern was apparent between the four treatment groups. The time-averaged treatment means for each PROMIS T-score outcome were not different.

CLINICAL STUDY PROTOCOL

Randomized multicenter phase 3 single-blind trial comparing the efficacy of corticosteroid control to mesenchymal stem cell preparations from autologous bone marrow concentrate (BMAC), adipose derived stem cells in the form of Stromal Vascular Fraction (SVF), and third party human mesenchymal stem cells manufactured from umbilical cord tissue for the treatment of unilateral Knee Osteoarthritis (OA).

Protocol Number: **M**ulticenter tr**I**al of stem ce**L**l th**E**rapy for o**S**teoarthritis (**MILES**)

Protocol Version: 9.0

Protocol Date: 22-Oct-2020

Sponsor-Investigator Name: Scott Boden, MD

Study Leadership:

Scott Boden, MD: Overall Study PI (Emory)

Hicham Drissi, PhD: Scientific Program Director (Emory)

Lora Black, RN, MPH: CRO Director (Sanford)

Krish Roy, PhD: Cell analytics center Core Director (Georgia Tech)

Joanne Kurtzberg, MD: Cord Blood Bank Director (Duke)

Michael Gottschalk, MD: Clinical Research Director (Emory)

Eric Wagner, MS, MD: Medical Monitor (Emory)

Sites:

**Emory University:**	Kenneth Mautner, MD
Sanford Health:	
**Fargo**	Benjamin Noonan, MD, MS
**Sioux Falls**	Chad Kurtenbach, MD
**Duke University:**	Blake Boggess, DO
**Andrews Institute:**	Joshua Hackel, MD

Responsibility Acknowledgement:

I have read this study protocol and agree to adhere to the requirements. I, or my designee, will provide copies of this study protocol and all pertinent information to the study personnel. I, or my designee, will discuss this material with them and ensure they are fully informed regarding the study interventions and the conduct of the study according to the Food and Drug Administration (FDA) Guidelines for Good Clinical Practices (GCP), the institutional policies and procedures, and pertinent individual country laws/regulations.

Investigator	**Date**

Sponsor Signature Page:

____ _______________________________

Scott Boden, MD
Sponsor-Investigator
Emory University

Date

Contents

1.0 Protocol Summary *10*

1.1 Synopsis 10

1.2 Schema 13

1.3 Schedule of Activities (SoA) 14

2.0 Introduction *16*

2.1 Study Rationale 16

2.2 Background 16

2.3 Benefit/Risk Assessment 18

2.3.1 Procedural-Related Risks 18

2.3.2 Mitigation of Risks 21

3.0 Objectives and Endpoints *22*

3.1 Primary Objective 22

3.2 Secondary Objectives 22

3.3 Exploratory Objectives 22

4.0 Study Design *22*

4.1 Overall study design 22

4.2 Scientific Rationale for Study Design 23

4.3 Enrollment 25

4.4 End of Study Definition 25

4.5 Study Stopping Rules 25

5.0 Study Population *25*

5.1 Eligibility Criteria 25

5.1.1 Inclusion Criteria 25

5.1.2 Exclusion Criteria 26

5.2 Screen Failures 27

6.0 Study Intervention *27*

6.1 Study Intervention(s) Administered: 28

6.1.1 Autologous Bone Marrow Concentrate (BMAC) 28

6.1.2 Adipose-derived Stromal Vascular Fraction (SVF) 28

6.1.3 Mesenchymal Stem Cells derived from Umbilical Cord Tissue (UCT) 28

6.1.4 Corticosteroid Injection 28

6.2 Measures to Minimize Bias: Randomization and Blinding 28

6.2.1 Randomization Methods 29

6.2.2 Implementation of Randomization and Blinding 29

6.3 Study Compliance ... 29
6.4 Concomitant Therapy ... 30
7.0 Subject Discontinuation/Withdrawal ... 30
7.1 Subject Discontinuation ... 30
7.2 Subject Withdrawal of Consent ... 30
7.3 Lost To Follow Up ... 30
7.4 Treatment failure ... 31
7.5 Discontinuation of the Clinical Study ... 31
8.0 Study Assessments and Procedures ... 31
8.1 Study Visit Assessments ... 31
8.1.1 Screening Visit (Visit 1) ... 31
8.1.2 Treatment Visit (Visit 2, Day 0) ... 31
8.1.3 Week 1 (Visit 3, Day 7) ... 32
8.1.4 Month 1 (Visit 4, Day 30) ... 32
8.1.5 Month 3 (Visit 5, Day 90) ... 32
8.1.6 Month 6 (Visit 6, Day 180) ... 33
8.1.7 Month 9 Phone Call (Visit 7, Day 270) ... 33
8.1.8 Month 12/ (Visit 8, Day 365) ... 33
8.1.9 Unscheduled Visits ... 33
8.1.10 End of Study Visit ... 33
8.2 Clinical Assessments and Procedures ... 33
8.2.1 Informed consent and HIPAA Authorization ... 33
8.2.2 Assignment of Screening Number ... 34
8.2.3 Inclusion/Exclusion Criteria ... 34
8.2.4 Demographics ... 34
8.2.5 Medical and Surgical History ... 34
8.2.6 Prior Medications ... 34
8.2.7 Concomitant Medications and Treatments ... 34
8.2.8 Physical Examination ... 34
8.2.9 Vital Signs ... 35
8.2.10 MRI ... 35
8.2.11 Patient Reported Outcomes ... 35
8.2.11.1 Visual Analog Pain Scale (VAS) ... 35
8.2.11.2 Euro-Quality of Life (EQ5D 3L) ... 35
8.2.11.3 Knee Injury and Osteoarthritis Outcome Score (KOOS) ... 35
8.2.11.4 Patient-Reported Outcomes Measurement Information System (PROMIS 29) ... 35
8.2.12 Adverse Event Monitoring ... 35
8.2.13 Physical Therapy ... 35
8.2.14 Cell Characterization ... 36
8.2.15 Local Laboratory Assessments ... 36
8.2.15.1 Hematology ... 36
8.2.15.2 Clinical Chemistry ... 36
8.2.15.3 Coagulation Assays ... 36

8.2.15.4 Immunogenicity Testing 36
8.2.16 Pregnancy Testing 36

8.3 Protocol Deviations 37

8.4 Adverse Event Reporting and Safety Monitoring 37
8.4.1 Adverse Events 37
8.4.2 Serious Adverse Events 37
8.4.3 AE and SAE Collection 37
8.4.4 Follow-up of AEs and SAEs 38
8.4.5 Adverse Event and Serious Adverse Event Reporting 38
8.4.6 Investigational New Drug (IND) Safety Reports 38
8.4.7 Unanticipated Problems (Emory IRB Requirement) 38

9.0 Statistical Considerations 39

9.1 Statistical Hypothesis 39

9.2 Sample Size Determination 39

9.3 Efficacy Analyses 40
9.3.1 Efficacy Variables 40
9.3.2 Primary and Secondary Efficacy Analyses 40
9.3.3 Efficacy Analyses across Subgroups 41

9.4 Safety Analyses 41
9.4.1 Trial Drug Exposure 41
9.4.2 Adverse Events 42
9.4.3 Continuous Monitoring of Specific Adverse Events 42
9.4.4 Analysis of Adverse Events 43

9.5 Interim Analyses and Data Monitoring 44

10.0 Ethical Considerations and Compliance with Good Clinical Practice 44

10.1 Statement of Compliance 44

10.2 Participating Centers 45

10.3 Informed Consent 45

10.4 Study Subject Confidentiality 45

10.5 Study Documentation and Case Report Forms (CRFs) 46

10.6 Monitoring of the Study 46

10.7 Direct Access to Source Data/Documents 47

10.8 Medical Monitor and Data Safety Monitoring Board (DSMB) 47

10.9 Publication Policy 48

10.10 Maintenance and Retention of Records 48

11.0 References 49

12.0 Appendices 50

12.1 Joint Rehab..50
12.2 Lab Value Reference Ranges 51

List of Abbreviations

ADP	Adipose Tissue
ADSC	Adipose Derived Stem Cells
AE/SAE/UP	Adverse Event/Serious Adverse Event/Unanticipated Problem
BMAC	Autologous Bone Marrow Concentrate
BMI	Body Mass Index
CDC	Centers for Disease Control
CFR	Code of Federal Regulations
CRF/eCRF	Case Report Form/electronic Case Report Form
CRO	Contract Research Organization
CSI	Corticosteroid Injections
DHHS	Department of Health and Human Services
DOD	Department of Defense
DSMB	Data Safety Monitoring Board
FDA	Food and Drug Administration
FCBP	Female of Child Bearing Potential
GCP	Good Clinical Practices
GEE	Generalized Estimating Equations
GMP	Good Manufacturing Practices
HIPAA	Health Insurance Portability and Accountability Act
ICF	Informed Consent Form
ICH	International Conference on Harmonization
IKDC	International Knee Documentation Committee
IND	Investigational New Drug
IRB	Institutional Review Board
KOOS	Knee Injury and Osteoarthritis Outcome Score
MAR	Missing at Random
MC3M	Marcus Center for Therapeutic Cell Characterization and Manufacturing
MedDRA	Medical Dictionary of Regulatory Activities
MRI	Magnetic Resonance Imaging
MSCs	Mesenchymal Stem Cells
NCI-CTCAE	National Cancer Institute-Common Terminology Criteria for Adverse Events
NIH	National Institutes of Health
NSAIDs	Non-Steroidal Anti-Inflammatory

OA	Osteoarthritis
OHRP	Office for Human Research Protections
PHI	Personal Health Information
PI	Principal Investigator
PRO	Patient Reported Outcomes
PRP	Platelet Rich Plasma
PSIS	Posterior Superior Iliac Spine
RCT	Randomized Controlled Trial
RPM	Revolutions per Minute
SAP	Statistical Analysis Plan
SAS	Statistical Analysis System
SoA	Schedule of Activities
SVF	Stromal Vascular Fraction
UCT	Umbilical Cord Tissue
UP	Unanticipated Problem
USA	United States of America
VA	Veteran's Administration
VAS	Visual Analog Scale

1.0 Protocol Summary

1.1 Synopsis

**Title of Study**	Randomized multicenter phase 3 single-blind trial comparing the efficacy of corticosteroid control to mesenchymal stem cell preparations from autologous bone marrow concentrate (BMAC), adipose derived stem cells in the form of Stromal Vascular Fraction (SVF), and third party human mesenchymal stem cells manufactured from umbilical cord tissue (UCT) for the treatment of unilateral Knee Osteoarthritis (OA).
**Study Objectives**	To identify a superior source of stem cells for the treatment of osteoarthritis and validate its advantages over corticosteroid injections as traditional gold standard treatment. The primary efficacy co-outcomes are: a change in the visual analog score (VAS) pain score and a change in the pain subsection of the knee injury and osteoarthritis outcome (KOOS) score. Secondary endpoints include total KOOS score, Magnetic Resonance Imaging (MRI) and additional patient reported outcomes (PROs).
**Study Design**	Multicenter prospective single-blinded randomized controlled clinical trial. The study will include a parallel design using a blocked central randomization scheme of 1:1:1:1. Subjects will be allocated to three different arms with a 3:1 assignment. Autologous bone marrow concentrate (n=120): corticosteroid (n=40), umbilical cord tissue (n=120): corticosteroid (n=40), and adipose derived SVF (n=120): corticosteroid (n=40).
**Eligibility Criteria**	**Inclusion Criteria:**<br>1. Age ≥40 but ≤70 years old<br>2. Males and Females<br>3. Recent knee radiograph of the targeted knee (Standing AP lateral and sunrise view)<br>4. Diagnosis of OA in the targeted knee (radiographic evidence of OA in the medial and/or lateral tibiofemoral compartment, which would include one or more osteophytes on a standard radiograph taken within 3 months). Subjects may have concomitant patellofemoral but must have grade II or higher medial or lateral tibiofemoral knee OA and the primary pain must be related to tibiofemoral arthritis.<br>5. Continued OA pain in the targeted knee despite conservative measures (per treating provider's discretion)<br>6. Average daily VAS ≥ 3<br>7. Kellgren-Lawrence system of Grade II, III, or IV<br>8. Females of childbearing potential (FCBP) only, must have a negative pregnancy test done at screening prior to enrollment. FCBP is defined as any premenopausal female unless they have had a surgical sterilization procedure such as, but not limited to, hysterectomy, oophorectomy, or tubal ligation<br>9. Women and men of child-producing potential must agree to use acceptable contraception methods for the duration of the trial such as birth control pills or condoms with spermicide

	**Exclusion Criteria:**<br>1. Clinically apparent tense effusion of the targeted knee<br>2. Significant valgus/varus deformities (+/- 10 degrees)<br>3. Viscosupplementation within 6 months in the targeted knee<br>4. Other Biologic Injection (PRP or stem cell) within 1 year in the targeted knee<br>5. Surgery in the targeted knee within the past 6 months (either open or scope)<br>6. Systemic or intra-articular injection of corticosteroids in any joint within 3 months before screening<br>7. Daily opioid use for the past three months<br>8. History of malignancy in the previous 5 years prior to study entry, with the exception of in-situ cancers treated only by local excision with curative intent.<br>9. History of, or ongoing, autoimmune disorder that requires treatment with an immunosuppressive medication<br>10. Active, suspected, or prior infection to the joint in the targeted knee<br>11. Part of a vulnerable population per Office for Human Research Protections (OHRP) definition (pregnant women and breast-feeding women, cognitively impaired, prisoners, etc.)<br>12. Unwilling to discontinue the use of Non-Steroidal Anti-Inflammatory (NSAID)s for 7 calendar days prior to the procedure<br>13. Unwilling to discontinue use of NSAIDS for 5 calendar days after procedure<br>14. History of bleeding disorders or inflammatory joint disease<br>15. Inability to hold anti-platelet therapy according to treating provider prior to procedure<br>16. Subject is, in the opinion of the investigator, unable to comply with the requirements of the study protocol or is unsuitable for the study for any reason.<br>17. Uncontrolled diabetes (determined by the treating investigator)<br>18. Subject has an active workers' compensation case in progress with targeted knee<br>19. Subject with insufficient amount of subcutaneous tissue, at the discretion of the treating investigator upon examination.<br>20. Hemoglobin less than 10g/dL at the time of screening<br>21. Leukocytes <3,000/µL; neutrophils <1,500/µL; lymphocytes <800/µL; platelets <100,000/µL at the time of screening<br>22. Diagnosis of liver disease as defined by alanine aminotransferase (ALT) >3x the upper limit of age-determined normal (ULN) or total bilirubin > 1.5x ULN<br>23. Subjects who have had greater than 3 corticosteroid injections in the targeted knee in the 12 months prior to screening or at the physician's discretion.<br>24. Subjects with a known diagnosis of osteoporosis<br>25. Subjects with anticipated use of systemic corticosteroids during the study period for treatment of a chronic medical condition
**Sample Size**	Total of 480 subjects. 120 subjects at each of the 4 institutions.
**Statistical Analysis**	The sample size calculations for the proposed intention-to treat, parallel-group, multicenter, randomized, controlled trial is based on improvements (from baseline to 1-year) in the visual analog scale (VAS) pain score and the knee injury and osteoarthritis outcome (KOOS) score. As the pain subsection of the KOOS is more sensitive and specific, using the full KOOS score as a proxy should provide ample power for the study.
**Study Treatment**	Subjects will be divided among three parallel arms for ease of blinding.<br>**Arm 1** includes randomization to bone marrow derived MSCs versus corticosteroid injection.<br>**Arm 2** includes randomization to adipose derived MSCs versus corticosteroid injection.<br>**Arm 3** includes randomization to umbilical cord tissue MSCs versus

	corticosteroid injection.
**Study Evaluations**	Follow-up exams will occur at intervals of 7 days, 1 month, 3 months, 6 months, and 9 months (telephone only), and 1 year.<br>Subjects will undergo a pre-injection MRI and a follow-up MRI at 6 and 12 months. Total of 3 MRIs per subject.<br>Subject will complete PROs at each time point.
**Study Period**	All randomized subjects will be followed in the study for approximately 1 year after treatment. If subjects continue to show improvement at the 1-year time frame, additional funding may be sought to provide ongoing treatment.

1.2 Schema

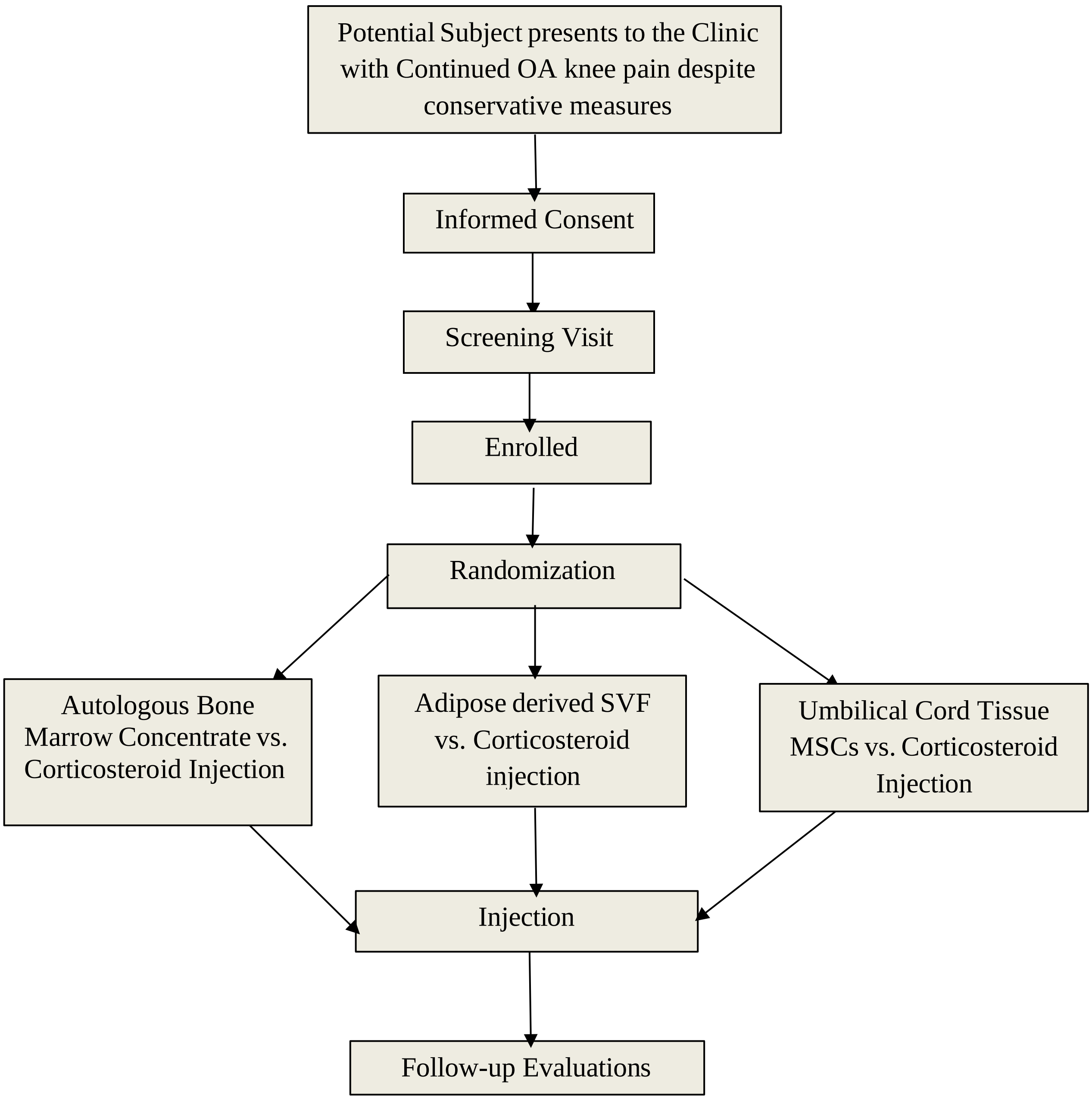
Potential Subject presents to the Clinic with Continued OA knee pain despite conservative measures
Informed Consent
Screening Visit
Enrolled
Randomization
Autologous Bone Marrow Concentrate vs. Corticosteroid Injection
Adipose derived SVF vs. Corticosteroid injection
Umbilical Cord Tissue MSCs vs. Corticosteroid Injection
Injection
Follow-up Evaluations

1.3 Schedule of Activities (SoA)

This study consists of the following phases:

	**Screening Visit**	**Treatment**	**Follow-Up**						**Unscheduled Visit/EOS**[jm]
**Days**	-30 days	0	7	30	90	180	270	365	UNS
**Scheduling Window (+/- days)**			+/- 1 day	+/-15 days	+/-15 days	+/-30 day	+/-30 days	+/-30 days	As needed
**Administrative Procedures**									
Informed Consent & HIPAA	X								
Randomization	X								
Inclusion/Exclusion Criteria	X								
Demographics & Medical/Surgical History	X								
Prior and Concomitant Medications and Treatments	X	X	X	X	X	X	X	X	X
Knee Osteoarthritis History	X								
**Clinical Procedures and Assessments**									
Focused Knee Assessment (knee & harvest site[k])	X	X[n]	X	X	X	X		X	X
Vital Signs	X	X	X	X	X	X		X	X
Phone Call							X		
MRI	X[e]					X		X	
PROs	X			X	X	X	X	X	
Adverse Events Monitoring	X	X	X	X	X	X	X	X	X
**Study Procedure/Treatment**									
Pre-Treatment Confirmation of Eligibility[p]		X							
Lipoaspirate procedure, Bone Marrow Aspiration, or UCT-MSCs Dispensing		X[d]							
Cell Processing[q]		X							
Product Release Criteria Testing[o]		X							
CSI Preparation[r]		X							
Arthrocentesis[f] & Knee injection		X							
**Cell Characterization (MC3M samples)**									
Sample Processing and Storage		X							

CONFIDENTIAL: The information contained within this document is legally privileged or otherwise exempt for unauthorized disclosure under applicable law

**Local Laboratory Assessments**									
CBC[ah]	X			X					X
Basic Metabolic Panel[ai]	X			X					X
PT/INR[ag]	X			X					X
Liver Function Tests[ai]	X			X					X
Immunogenicity Testing (UCT arm only)[cl]				X				X	
Pregnancy Test	X[b]	X[b]							

[a] Subject will be referred to their primary care provider if any abnormalities (not already known to the subject or in the medical history) are discovered from the safety labs and deemed clinically significant by the treating investigator.

[b] FCBP: Obtain urine or serum pregnancy test.

[c] Immunogenicity testing which consists of a panel reactive antibodies (PRA), will be performed on the recipients of the umbilical cord tissue. All subjects in the UCT arm will have a blood draw to maintain subject blinding.

[d] Subjects randomized to Arm 1 will undergo the bone marrow aspiration. Subjects randomized to Arm 2 will undergo the lipoaspirate procedure. UCT-MSCs dispensing will only occur for subjects randomized to Arm 3 / UCT treatment group.

[e] Only subjects that meet all eligibility criteria will undergo the Screening MRI

[f] Investigator will aspirate synovial fluid prior to knee injection when possible (physician's discretion). Collected synovial fluid will be sent to MC3M for testing (details in study manuals).

[g] PTT may be ordered if deemed appropriate by the treating investigator

[h] CBC should include a platelet count

[i] Basic metabolic panel and Liver function panel may be substituted for a Comprehensive metabolic panel (physician's discretion) as long as the test includes an ALT and Total Bilirubin

[j] Assessments completed during an Unscheduled Visit will be at the discretion of the treating investigator based on the reason for visit and examination of the subject. Laboratory assessments may be ordered at the discretion of the treating investigator.

[k] Observation of the harvest site will be completed at the 7-day follow-up and 30-day follow-up visits. This will only be done for subjects on Arm 1 and Arm 2.

[l] Please refer to the Operations Manual for specific results required for this testing

[m] End of study visit: Assessments required will be at the discretion of the treating investigator in order to ensure subject safety. The EOS visit would only occur in the event the study is discontinued early (see Section 7.5)

[n] Optional, at the discretion of the treating investigator

[o] Refer to the Operations Manual for Arm-specific product release criteria requirements.

[p] Treating Investigator must complete the Pre-Treatment Confirmation of Subject Eligibility form PRIOR to procedure or UCT dispensing.

[q] Only subjects randomized to Arm 1 or Arm 2.

[r] Only subject randomized to receive the CSI treatment in any of the 3 arms.

CONFIDENTIAL: The information contained within this document is legally privileged or otherwise exempt for unauthorized disclosure under applicable law

2.0 Introduction

2.1 Study Rationale

Primary osteoarthritis is a debilitating disease characterized by (i) extensive damage to the joints, (ii) excruciating pain leading to loss of activity and depression. Osteoarthritis is a significant clinical dilemma in the USA and around the world. Despite advances in diagnosis and relatively efficient control of nociception, to date, the quest for the development of a disease modifying osteoarthritis drug has proven unsuccessful. The consequence of this has been to employ ineffective non-surgical procedures until extensive wearing of the cartilage has forced surgeons to perform expensive and risky joint replacement therapies.

The economic burden [4] for treatment and lost productivity caused by osteoarthritis amounts to 60 billion annually in the US alone. According to the Centers for Disease Control (CDC), arthritis currently affects more than 20 million Americans.

With an ever-growing aging population, debilitating pain, loss of muscle function and decreased physical activity further enhance associated costs of care to treat diabetes, depression and other secondary consequences of osteoarthritis. A lesser-recognized consequence of such chronic ailment is the emotional burden of family members who care for osteoarthritic patients.

2.2 Background

The potential of mesenchymal stem cells to inhibit inflammation while promoting healing makes them amenable for the treatment of various ailments ranging from cancer to genetic diseases. In orthopedic practice, autologous stem cell injections are performed to alleviate the pain associated with osteoarthritis. Thus, at all recruiting sites, physicians have led multiple studies geared towards understanding the advantages and disadvantages of using autologous stem cells for the treatment of osteoarthritic joints. A thorough search of the orthopedic literature yielded limited injectable cell data, most of which describe case series. A serious gap in knowledge remains whether the currently used cellular treatments are beneficial in the long term and if one cell therapy outperforms another. Finally, our survey of the clinical trials in the continental U.S. failed to show any head to head comparisons of the three-proposed mesenchymal derived cell lines.

Although the use of various stem cell preparations for knee OA has become increasingly prevalent, well-designed studies with conclusive proof of comparative effectiveness and identification of the optimal cell source and "dose" have not been performed. Thus, we propose the first Randomized Clinical Trial (RCT) comparing three types of cellular treatments in one study. Our exhaustive outcomes, which include Patient Reported Outcomes (PROs), radiographic data analyses (cartilage and joint health), and high level cell characterization has never before been described and will fill in a significant gap in

knowledge. Finally, we will integrate our clinical studies with state of the art evaluations of the cells that will be injected. This blend of clinical and translational research will be highly impactful and will help transform the current clinical practice of cellular therapy for orthopedic applications and beyond. This information will substantially advance the science behind stem cell usage in orthopedics and hopefully provide support to justify broader usage and approval as an alternative to surgical "salvage" intervention.

Autologous Mesenchymal Stem Cells (MSCs) have been used for more than two decades to treat orthopedic conditions. The most popular form of MSC therapy has been through the use of Autologous Bone Marrow Concentrate (BMAC). The rationale is that when a sample of Bone Marrow Aspirate (BMA) is collected and the components that are not beneficial to the joint are filtered out (i.e. red blood cells, neutrophils, etc.) the remaining concentrate (MSCs, platelets, Interleukins, etc.) can have a “healing” effect on the environment in which it is injected. This healing affect is mediated mostly through paracrine functions of the MSCs as well as potential differentiation into healthy tissue. Over the last 10 years since the FDA has stopped allowing culture and expansion of cells in the laboratory, the use of same day MSC treatments through BMAC has become more popular. What is still unknown is how effective is BMAC for treating orthopedic conditions compared to other MSC procedures. Also, it is unknown what are the most important components of the BMAC mixture that could aid patients suffering from orthopedic conditions, particularly osteoarthritis.

Bone Marrow Aspiration (BMA) procedures have been done for many years now with few reported complications. A large case series of complications following BMA procedures was published by Centeno et al in 2015 and reveals that of 3012 procedures, there were a total of 325 Adverse Events (AE) reported. 183 of these AE’s were related to pain at the harvest site or the injection site due to the patients’ underlying degenerative joint disease. The other reported complications were mostly due to procedural risk that would be present with any percutaneous procedure, namely infection either locally or deeper in the bone marrow. There were seven reported neoplasms during the study follow up which is actually lower than the incidence of neoplasms in the general population so is largely thought to not be at all related to the BMA procedure. The MSCs themselves, because of their known immuno-privileged status, are not thought to confer any additional risk to the subject other than potential increased pain at the site of injection from the pro-inflammatory cytokines that are known to be in the concoction.

Umbilical cord tissue (UCT) is less well defined. However, as reported in NCT02176317, the safety and tolerability profile of autologous CB infusion in ASD was excellent. No serious adverse events were reported, and adverse events, in general, were sparse. Three children had mild allergic reactions associated temporally with the infusions, consisting of cough and hives during infusion for 1 child and cough post-infusion for 2 children. Also, one child was noted to be more irritable for 2 days post-infusion. No subjects discontinued prematurely from the study due to adverse events.

Lipoaspirate is obtained via liposuction, or the removal of adipose tissue via a suction method, is a common cosmetic procedure performed by plastic surgeons. Depending on the liposuction location and indication, 100cc to 2,000cc of lipoaspirate can be safety removed from a subject during a single liposuction procedure. Through a tissue process which includes washing and centrifuging, the cellular components can be extracted as a cell pellet, which is also known as Stromal Vascular Fraction (SVF). This cellular product is thought to contain adipose tissue-derived stem cells as well as leukocytes, erythrocytes and other cell types. However, numerous pre-clinical studies that have analyzed SVF have shown that it contains a heterogeneous cell population, thus, making its precise cellular components unclear. Furthermore, the fate of these cellular components can vary significantly; the age of the adipose tissue donor, the type and localization of the adipose tissue, and cellular isolation process may affect the cellular analysis outcome.

Adipose tissue has been found to have a large amount of mesenchymal stem cells versus that in bone marrow. These cells are currently being used in a variety of clinical research studies within the regenerative medicine field. The cellular pellet, SVF, can be easily maintained and expanded in culture, showing stable growth and proliferation rates [8].

2.3 Benefit/Risk Assessment

2.3.1 Procedural-Related Risks

Subjects may experience pain and/or discomfort during and after the bone marrow and liposuction procedures despite the local anesthesia.

Bone Marrow Aspiration (Arm/Group 1)

Less Common (10-20%)

- Blood pressure may get low and cause symptoms such as dizziness, lightheadedness, confusion, or blurred vision [5]

Rare (1-10%)

- Bleeding
- Hematoma (bleeding under skin)
- Risk of infection at the needle insertion sites

Liposuction Procedure (Arm/Group 2)

Less Common (10-20%)

- Bruising
- Hematoma (bleeding under skin)
- Change in skin sensation that may persist
- Swelling
- Fluid accumulation

Rare (1-10%)

- Local anesthesia risks
- Damage to deeper structures such as nerves, blood vessels, muscles, lungs and abdominal organs (<1%)
- Deep vein thrombosis
- Cardiac and pulmonary complications
- Infection
- Irregular contours or asymmetries
- Irregular pigmentation
- Need for revision surgery
- Persistent swelling
- Rippling or loose skin, worsening cellulite
- Poor wound healing

SVF and BMAC Cell Mixture Injection

The exact risks from the cell application are not entirely known. However, literature shows that using your own cells for a clinical therapy does not cause inflammation, rejection or tumor growth.

UCT Mesenchymal Stem Cell Injection

Below are risks associated with UCT MSCs given intravenously for other conditions. To date, there are no reported risks with knee injections of UCT MSCs.

- Transient headache
- Transient fever
- Allergic reaction

Knee Injection (All Arms/Groups)

Rare (1-10%)

- Pain at the site of injection
- Bleeding
- Infection
- Swelling at the site of injection
- Granulomatous inflammation of the synovium
- Saphenous Neuropathy
- Aseptic acute arthritis
- Septic Arthritis
- Pigment changes
- Mild pain or swelling at the site of injection may occur in up to 20% of subjects
- Severe local inflammation, warmth, and joint effusion are rare [6]

- Allergic reaction (UCT)

Steroid Injection (All Arms/Groups)

Less common (10-20%)
- Bruising at the injection site

Rare (1-10%)
- Death of nearby bone
- Joint infection
- Nerve damage
- Thinning of skin and soft tissue around the injection site
- Temporary flare of pain and inflammation in the joint
- Tendon weakening
- Lightening of skin surrounding the injection site
- Redness at the injection site
- Swelling at the injection site
- Bleeding at the injection site
- Infection
- Calcium deposits on the tendon site
- Allergic reaction

Epinephrine (Arm/Group 2)

Rare (1-10%)
- The feeling of a “racing heart”
- Temporary skin discoloration at the site of injection
- Sweating
- Nausea and vomiting
- Pale skin
- Feeling short of breath
- Dizziness
- Weakness or tremors
- Headache
- Feeling nervous or anxious

Lidocaine (All Arms/Groups)

Rare (1-10%)
- Tingling
- Prolonged numbness
- Temporary skin discoloration
- Mild bruising, redness, itching or swelling where the medication was injected
- The feeling of “pins” and needles
- Feeling anxious, shaky, dizzy, restless, or depressed
- Drowsiness, vomiting, ringing in your ears
- Feeling like you may pass out

Oral Percocet (optional as needed)

Less common (10-20%)
- Dizziness
- Drowsiness
- Feel like throwing up
- Feeling faint
- Head Pain
- Stomach cramps

Rare (1-10%)
- Itching
- Not feeling well
- Dry mouth
- Excessive sweating
- Incomplete or infrequent bowel movements

Oral Valium (optional as needed)

Rare (1-10%)
- Memory problems
- Drowsiness, tired feeling
- Dizziness, spinning sensation
- Feeling restless or irritable
- Muscle weakness
- Nausea, constipation
- Drooling or dry mouth, slurred speech
- Blurred vision, double vision
- Mild skin rash, itching
- Confusion
- Depression

Blood draw

Rare (1-10%)
- Bleeding
- Pain
- Bruising
- Redness, swelling of the vein
- Infection
- Fainting

MRI

May cause a metal object in your body to move out of place and cause serious injury or stop working properly [7]

2.3.2 Mitigation of Risks

Standard subject safety precautions will be employed for the procedures listed above, per institutional guidelines and best practice.

3.0 Objectives and Endpoints

3.1 Primary Objective

The primary efficacy co-outcomes are: a change in the visual analog scale (VAS) pain score and a change in the pain subsection of the knee injury and osteoarthritis outcome (KOOS) score.

3.2 Secondary Objectives

Secondary outcomes include change in total KOOS score, EuroQuality of Life (EQ5D 3L) and Patient-Reported Outcomes Measurement Information System (PROMIS 29).

Changes in MRI biomarkers of cartilage and joint health between the four treatment groups will be compared.

3.3 Exploratory Objectives

To provide deep and broad characterization of all cells used in this trial, harmonize and standardize analytical and processing parameters across the different clinical sites and eventually, as patient outcome data become available, to apply quantitative advanced modeling tools to identify predictive signatures of cell properties that correlate with efficacy and safety. When feasible, synovial fluid will be aspirated for cell characterization.

4.0 Study Design

4.1 Overall study design

We propose a multicenter prospective single-blinded randomized controlled clinical trial to test the safety and efficacy of autologous bone marrow (BMAC), adipose tissue (ADP), and third party human mesenchymal stem cells manufactured from umbilical cord tissue (UCT) to a current standard, corticosteroid injections (CSI), for the treatment of Knee Osteoarthritis. The study will include a parallel design using a blocked central randomization scheme of 1:1:1:1 (BMAC:ADP:UCT:CSI).

Subjects, who have provided written informed consent and are determined to be eligible, will be randomized to one of the following arms and treatments.

Arm 1 includes randomization to bone marrow derived MSCs versus corticosteroid injection.

Arm 2 includes randomization to adipose derived MSCs versus corticosteroid injection.

Arm 3 includes randomization to umbilical cord tissue MSCs versus corticosteroid injection.

Subjects randomized to Arm 1 or Arm 2 will undergo a procedure (bone marrow aspiration or liposuction) but will only receive one of the possible injections. For instance, subjects randomized to the control portion of Arm 1 will undergo a bone marrow aspiration but will receive a blinded corticosteroid injection. The bone marrow aspirate will be processed and utilized for cell characterization.

The corticosteroid cohort will serve as the control group for the trial. An injection of normal saline and Depomedrol will be used in a standard agreed upon preparation as described in the Operations Manual.

Two of the MSC preparations, adipose tissue and bone marrow, will be derived from autologous tissues and the third will be derived from a third party, unrelated allogeneic donor human umbilical cord tissue. The autologous products will be processed at the point of care and delivered without cryopreservation.

The allogeneic cord tissue MSC product will be manufactured and cryopreserved at the GMP cell manufacturing laboratory at Duke University Medical Center. This product will be shipped to the study sites in the cryopreserved state and thawed immediately prior to administration at the site. In all cases, the product administered to the subject will be characterized with cell counts, viability, sterility cultures, flow cytometry or mass cytometry and functional assays.

In order to accurately assess the efficacy of these treatments, routine follow-up examinations will be necessary. Follow-up exams will occur at intervals of 1 week, 1 month, 3 months, 6 months, 9 months (telephone only) and 1 year. After completing the screening and consent portion of the trial, the subjects will undergo a pre-injection MRI and then an additional MRI at 6 and 12 months. Additionally, subjects will fill out PROs at each time point.

The Marcus Center for Therapeutic Cell Characterization and Manufacturing (MC3M) at Georgia Institute of Technology will serve as a core cell analytics center for this clinical trial. MC3M's goal is to provide deep and broad characterization of all cells used in the trial, including synovial fluid, harmonize and standardize analytical and processing parameters across the different clinical sites and eventually, as patient outcome data become available, the MC3M will apply quantitative advanced modeling tools to identify predictive signatures of cell properties that correlate with efficacy and safety.

4.2 Scientific Rationale for Study Design

The current treatment of osteoarthritis relies on joint stability and non-operative control of inflammation. Oral non-steroidal anti-inflammatory (NSAID) options remain the initial line of treatment. When oral NSAIDs have failed, treatments with intra-articular

injectable corticosteroids for local pain management have proven successful for a short period of time, though evidence exists that their long-term use can be detrimental.

Other non-surgical treatment options include the injection of hyaluronic acid or other gels and biologics such as platelet rich plasma. While these treatments do provide pain relief, their precise mechanism of action is not known.

Joint arthroplasty is a definitive and successful surgical intervention typically offered to age appropriate patients after other less invasive measures have failed. However, this is substantial surgical intervention and possesses the inherent risks of any such procedure. Concern also exists regarding the longevity of knee arthroplasty, particularly in the younger, more active population.

Cell therapies have been increasingly employed to treat osteoarthritis, which include various forms of autologous (harvested from the same patient's body) mesenchymal stem cell injections. However, while this procedure has shown better outcomes than other non-surgical treatments, questionable reproducibility and the limited supply of stem cells limits the potential improvements to this valuable technique.

A large case series (41 subjects, 75 knees) with different biologics injections, most of which were autologous bone marrow concentrate (BMAC) + adipose derived stromal vascular fraction (SVF) were injected into the knee joint. Visual analog score (VAS), knee injury and osteoarthritis score (KOOS), international knee documentation committee (IKDC) and Lysholm knee Scores (patient reported outcome measures, PRO's) were all improved in the treated group [1]. ***Better outcomes were noted in those with lower degrees of osteoarthritis.***

Recently, a well-designed small clinical (proof of concept) trial was done with 2-year follow-up, evaluating different dosages of adipose derived SVF which were cultured and injected for the treatment of knee osteoarthritis. What made this study interesting was that it examined small, medium, and high doses of MSCs and evaluated their effects using MRI, arthroscopy, and clinical outcomes [2]. ***However, there was no control group and the study did not have a large number of subjects.***

Another study investigated a control group (saline) compared to same day BMAC preparation (non-expanded). The cells were characterized and the injections were done on bilateral knees with one knee getting the control and one getting the BMAC. The results showed marked improvement in symptoms but it was not statistically different between the two groups secondary to under powering of the study [3]. ***The fact that saline control also showed a good outcome indicates that such study was not well controlled and more rigorous studies with an unbiased approach throughout several recruiting sites is needed.***

There is an urgent need to establish which cell based therapy will yield sustained and reproducible treatment for osteoarthritis. We believe that the cell source is a key

determinant of the success of the MSC injections into the knee joint to alleviate pain and immediately enhance function and even halt the natural progression of the diseases.

4.3 Enrollment

Subjects will be enrolled after the investigator has completed the following: confirm the subject has signed the informed consent and Health Insurance Portability and Accountability Act (HIPAA) Authorization, all screening assessments have been completed, and confirm that the subject has met all the inclusion criteria and none of the exclusion criteria.

Randomization can occur at any time after confirmation that the subject meets eligibility, but should occur within 30 days of the date of informed consent form signing. If the period is more than 30 days, an updated informed consent process must take place.

Enrollment of subjects is expected to last approximately 13 months in order to obtain the numbers needed. An additional three months has been incorporated into the study to allow for unanticipated delays and potential protocol changes.

4.4 End of Study Definition

A subject is considered to have completed the study if he/she has completed all phases of the study including the one-year follow-up visit. The end of the study is defined as the date of the last visit of the last subject in the study shown in the Schedule of Activities for the last subject in the trial.

Final data analysis will occur following completion of study activities.

4.5 Study Stopping Rules

If one of the following safety issues occurs, further enrollment will be halted until the Medical Monitor, Dr. Eric Wagner, reviews all available data and makes recommendations as to the next steps:

- Any potential related serious adverse event (SAE).
- Other findings that, at the discretion of the study team, DSMB, investigator and/or Medical Monitor, indicate that the study should be halted.

5.0 Study Population

5.1 Eligibility Criteria:

The following is a list of stringent inclusion and exclusion criteria that is designed to reduce confounding and conflicting variables while studying the effect of biologic injections for knee osteoarthritis.

5.1.1 Inclusion Criteria

1. Age ≥40 but ≤70 years old
2. Males and Females

3. Recent knee radiograph of the targeted knee (standing AP lateral and sunrise view)
4. Diagnosis of OA in the targeted knee (radiographic evidence of OA in the medial and/or lateral tibiofemoral compartment, which would include one or more osteophytes on a standard radiograph taken within 3 months). Subjects may have concomitant patellofemoral but they must have grade II or higher medial or lateral tiobiofemoral knee OA and the primary pain must be related to the tibiofemoral arthritis.
5. Continued OA pain in the targeted knee despite conservative measures (per treating provider's discretion)
6. Average daily VAS ≥3
7. Kellgren-Lawrence system of Grade II, III, or IV
8. Females of childbearing potential (FCBP) only, must have a negative pregnancy test done at screening prior to enrollment. FCBP is defined as any premenopausal female unless they have had a surgical sterilization procedure including, but not limited to, hysterectomy, tubal ligation or oophorectomy.
9. Women and men of child-producing potential must agree to use acceptable contraception methods for the duration of the trial such as birth control pills or condoms with spermicide.

5.1.2 Exclusion Criteria

1. Clinically apparent tense effusion of the targeted knee
2. Significant valgus/varus deformities (+/- 10 degrees)
3. Viscosupplementation within 6 months in the targeted knee
4. Other Biologic Injection (PRP or stem cell) within 1 year in the targeted knee
5. Surgery in the targeted knee within the past 6 months (either open or scope)
6. Systemic or intra-articular injection of corticosteroids in any joint within 3 months before screening
7. Daily opioid use for the past three months
8. History of malignancy in the previous 5 years prior to study entry, with the exception of in-situ cancers treated only by local excision with curative intent.
9. History of, or ongoing, autoimmune disorder that requires treatment with an immunosuppressive medication
10. Active, suspected, or prior infection to the joint in the targeted knee
11. Part of a vulnerable population per Office for Human Research Protections (OHRP) definition (pregnant women and breast-feeding women, cognitively impaired, prisoners, etc.)
12. Unwilling to discontinue the use of Non-Steroidal Anti-Inflammatory (NSAID)s for 7 calendar days prior to the procedure

13. Unwilling to discontinue use of NSAIDS for 5 calendar days after procedure
14. History of bleeding disorders or inflammatory joint disease
15. Inability to hold anti-platelet therapy according to treating provider prior to procedure
16. Subject is, in the opinion of the investigator, unable to comply with the requirements of the study protocol or is unsuitable for the study for any reason.
17. Uncontrolled diabetes (determined by the treating investigator)
18. Subject has an active workers' compensation case in progress with targeted knee
19. Subject with insufficient amount of subcutaneous tissue, at the discretion of the treating investigator, upon examination
20. Hemoglobin less than 10g/dL at the time of screening
21. Leukocytes <3,000/µL; neutrophils <1,500/µL; lymphocytes <800/µL; platelets <100,000/µL at the time of screening
22. Diagnosis of liver disease as defined by alanine aminotransferase (ALT) >3x the upper limit of age-determined normal (ULN) or total bilirubin > 1.5x ULN
23. Subjects who have had greater than 3 corticosteroid injections in the targeted knee in the 12 months prior to screening or at the physician's discretion.
24. Subjects with a known diagnosis of osteoporosis.
25. Subjects with anticipated use of systemic corticosteroids during the study period for treatment of a chronic medical condition.

5.2 Screen Failures

Screen failures are defined as subjects who consent to participate in the clinical study but are not subsequently enrolled. A minimal set of screen failure information is required to ensure transparent reporting of screen failure subjects. Minimal information should include demography, screen failure details, eligibility criteria, and any serious adverse event (SAE).

6.0 Study Intervention

Bone marrow aspiration will be completed on all subjects randomized to Arm 1. Small volume lipoplasty will be completed on all subjects randomized to Arm 2.

Prior to the knee injection, all samples will undergo point of care testing. If the BMAC, SVF final products or the UCT MSCs do not meet the final product acceptance criteria, as outlined in the Operations Manual, the subject will be discontinued from the study. The study team will maintain contact with the subject during the subsequent 30 days including in-person visit to assess any potential AEs and SAEs that the subject may have experienced after the study

treatment procedure. Subject will be withdrawn after 30 days if all study-related AEs are resolved.

All injections will be done into the knee joint via ultrasound guidance using a standard approach as described in the Operations Manual. Prior to the study injection, the treating investigator may aspirate excess synovial fluid for future cell characterization testing at MC3M (see Section 8.2.14).

6.1 Study Intervention(s) Administered:

6.1.1 Autologous Bone Marrow Concentrate (BMAC)

Bone Marrow Aspiration Concentrate (BMAC) is a standard Orthobiologic injection done for patients with knee osteoarthritis. This is a one step, same day procedure that involves harvesting of bone marrow aspirate (BMA) from the posterior superior iliac spine (PSIS) and then following centrifugation in an FDA approved device (EmCyte GenesisCS Pure BMAC®-60 ml), will be injected back into the knee joint for therapeutic effect. Details of the procedure are described in the Operations Manual.

6.1.2 Adipose-derived Stromal Vascular Fraction (SVF)

Lipoaspirate is obtained from a mini-lipoaspirate procedure. The lipoaspirate will then be enzymatically digested to produce a stromal vascular fraction (SVF) that will be injected into the knee joint. Details of the procedure are described in the Operations Manual.

6.1.3 Mesenchymal Stem Cells derived from Umbilical Cord Tissue (UCT)

Umbilical Cord Tissue has not been extensively studied for injections in knee Osteoarthritis. For this study, each study site will be provided with cryopreserved doses of cord tissue MSCs. These MSCs were cyopreserved at P2 culture in plasmalyte A + 5% human serum albumin in 5 finger cryobags containing 20 million cells in 4 mL and stored under liquid nitrogen until shipment to the study sites. Cells will be transported in a dry shipper and thawed at the point of care. The dose of MSCs will be thawed, aspirated from the cryobag into a sterile syringe and directly injected into the study subject's knee. Details of the procedure are described in the Operations Manual.

6.1.4 Corticosteroid Injection

A standard and routine procedure in the care of subjects with Osteoarthritis is an injection of corticosteroids into the knee joint. Corticosteroids have been shown to be effective for short-term relief of knee pain associated with Osteoarthritis and will serve as our standard of care procedure to compare to our cellular treatments. Details of the procedure are described in the Operations Manual.

6.2 Measures to Minimize Bias: Randomization and Blinding

The proposed clinical trial will allow for the unbiased evaluation of the efficacy of adult autologous versus third party stem cells and compare that efficacy to a gold standard treatment (e.g. corticosteroids).

6.2.1 Randomization Methods

Treatment assignments will be generated using a pseudo-random-number generator with randomly permuted blocks. This method serves to balance the treatment group assignments over the course of the study to ensure that the desired number of subjects will be allocated to each of the treatment groups at any time point during the randomization period. The statistical method for implementation of the randomization plan will be detailed in the Statistical Analysis Plan (SAP). Briefly, after obtaining informed consent, all eligibility confirmation data will be entered into the RAVE EDC database by the delegated study staff. If the subject meets all eligibility criteria, computer randomization will be performed and the coordinator will be provided with an audit trail of the process including a verifiable link between the unique study ID and treatment assignment. The application will be password protected with access restricted to designated MILES personnel.

6.2.2 Implementation of Randomization and Blinding

Subjects randomized to Arm 1 or Arm 2 will undergo a procedure (bone marrow aspiration or liposuction) but will only receive one of the possible injections. For instance, subjects randomized to the control portion of Arm 1 will undergo a bone marrow aspiration but will receive a blinded corticosteroid injection. The bone marrow aspirate will still be processed and used to help quantify cell characteristics. Subjects randomized to Arm 3 will not undergo the procedures mentioned above. They will be blinded and receive either the stem cell injection or the corticosteroid injection.

Four hundred eighty subjects will be randomly assigned to treatment in this multicenter trial. Subjects at each clinical site will be randomized to ensure the trial is single-blind with a 1:1 allocation ratio across the four treatment groups.

One hundred twenty subjects will be randomized at each of 4 clinical sites. Arm 1: Forty subjects will be randomized with a 3:1 allocation ratio (bone marrow derived MSCs versus corticosteroid injection; 30:10). This same implementation plan will be used to randomize 40 subjects to Arm 2 (adipose derived MSCs versus corticosteroid injection) with a 3:1 allocation ratio and to randomize 40 subjects to Arm 3 (umbilical cord tissue MSCs versus corticosteroid injection) with a 3:1 allocation ratio.

6.3 Study Compliance

Subjects will be contacted regularly by study team members with appointment reminders to aid in compliance with the protocol. Subjects will be encouraged to contact the investigator or study staff in between follow-up visits to report AEs and SAEs in a timely manner.

6.4 Concomitant Therapy

Concomitant therapy will not be utilized during the study as described in sections 5.1 and 5.2. Any interventions the subject may require after the study injection, will be monitored and recorded during follow-up visits. Concomitant therapies such as anti-inflammatories, pain medication, injections and surgery will be recorded on the Concomitant Medications and Treatments log.

7.0 Subject Discontinuation/Withdrawal

7.1 Subject Discontinuation

A subject must be discontinued from the trial for any of the following reasons:

- Unacceptable adverse experience
- Investigator's decision to withdraw the subject for safety, behavioral, compliance, or administrative reasons
- If the BMAC, SVF final products or UCT MSCs do not meet final product acceptance criteria outlined in the Operations Manual

For those subjects who discontinue participation early, each subject will be followed for serious adverse events (90 days) that are ongoing at the time of subject discontinuation. Monitoring of the serious adverse event may be done by telephone per treating investigator discretion. All AEs potentially related to study participation will be followed until resolution prior to study completion or early termination of subject. Open AEs that are unrelated to the study participation will not be followed beyond study completion or subject's early termination.

7.2 Subject Withdrawal of Consent

The investigator/treating physician will make every reasonable effort to keep each subject on study. A subject may withdraw from the study at any time at his/her own request without penalty, at any time during the study. If the subject withdraws consent for disclosure of future information, the sponsor may retain and continue to use any data collected before such a withdrawal of consent. All subject samples that were obtained prior to subject withdrawal will NOT be destroyed and future laboratory testing will be performed.

7.3 Lost To Follow Up

All reasonable efforts must be made to locate subjects to determine and report their ongoing status. This includes follow-up with persons authorized by the subject. Lost to

follow-up is defined by the inability to reach the subject after a minimum of three documented phone calls, faxes, or emails as well as lack of response by subject to one registered mail letter. All attempts should be documented in the subject's medical record. Should the subject continue to be unreachable, he/she will be considered to have withdrawn from the study.

If it is determined that the subject has died, the site will use permissible local methods to obtain the date and cause of death.

7.4 Treatment Failure

In order to reduce bias, if subjects require concomitant therapy (i.e. other injections), pain scores and subsequent treatment information will continue to be collected through the 1-year follow up after a subject is declared a treatment failure.

7.5 Discontinuation of the Clinical Study

The study may be prematurely discontinued by the Sponsor if deemed necessary for any reason. If for any reason the study is discontinued early, all subjects should complete the End of Study visit and be followed for 90 days for SAEs that were ongoing at the End of Study visit.

8.0 Study Assessments and Procedures

Subjects will have follow-up exams at 1 week, 1 month, 3 months, 6 months, 9 months (telephone only) and 1 year. After subjects complete the screening and consent portion of the trial, the subjects will undergo pre-injection MRI and then an additional MRI at 6 and 12 months. Additionally, subjects will fill out PROs at each time point.

8.1 Study Visit Assessments

8.1.1 Screening Visit (Visit 1)

- Administer informed consent and HIPAA Authorization
- Collect demography
- Collect medical and surgical history
- Collect prior and concomitant medications
- Collect Knee Osteoarthritis History
- Review inclusion and exclusion criteria
- Focused knee assessment
- Perform vital signs
- Knee MRI (only if the subject is eligible for the study)
- Collect subject PROs
- Perform pregnancy test (on female subjects of childbearing potential)
- Blood Collection (safety labs)
- Once all screening assessments are complete and eligibility has been confirmed by the treating investigator, the subject may be randomized.
- Schedule the subject for the appropriate Treatment Visit procedure based on randomization

8.1.2 Treatment Visit (Visit 2, Day 0)

- Collect concomitant medications
- Focused knee assessment (if required, at the discretion of the treating investigator)
- Perform vital signs
- Assess adverse events
- Perform pregnancy test (on female subjects of childbearing potential)
- Confirm subject eligibility
- Study procedures **(depending on subject randomization)**
    1. Arm 1-BMAC Treatment: bone marrow aspiration, cell processing and product release criteria testing
    2. Arm 1-CSI Treatment: bone marrow aspiration, cell processing, product release criteria testing and CSI preparation
    3. Arm 2-SVF Treatment: lipoaspirate procedure, cell processing, and product release criteria testing
    4. Arm 2-CSI Treatment: lipoaspirate procedure, cell processing, product release criteria testing and CSI preparation
    5. Arm 3-UCT Treatment: dispense UCT-MSCs, dose preparation, and product release criteria testing
    6. Arm 3-CSI Treatment: CSI preparation
- Perform arthrocentesis and knee injection
- Process and store samples for cell characterization (details are described in the Laboratory Manual)
- Home physical therapy program to begin post procedure day 8, see Appendix 12.1. Instructions for at home physical therapy will be provided to the subject by the study staff.

8.1.3 Week 1 (Visit 3, Day 7)

- Collect concomitant medications and treatments
- Focused knee assessment, including observation of knee injection site
- Observation of harvest sites (subjects in Arm 1 and Arm 2)
- Perform vital signs
- Assess adverse events
- Review progress of the at home physical therapy plan and encourage compliance

8.1.4 Month 1 (Visit 4, Day 30)

- Collect concomitant medications and treatments
- Focused knee assessment, including observation of knee injection site
- Observation of harvest sites (subjects in Arm 1 and Arm 2)
- Perform vital signs
- PROs
- Assess adverse events
- Blood Collection (safety labs)
- Immunogenicity testing (Arm 3 only)
- Review the subject’s at home physical therapy log for compliance

8.1.5 Month 3 (Visit 5, Day 90)

- Collect concomitant medications and treatments
- Focused knee assessment
- Perform vital signs
- PROs
- Assess adverse events

8.1.6 Month 6 (Visit 6, Day 180)

- Collect concomitant medications and treatments
- Focused knee assessment
- Perform vital signs
- MRI
- PROs
- Assess adverse events

8.1.7 Month 9 Phone Call (Visit 7, Day 270)

- Collect concomitant medications and treatments
- PROs
- Assess adverse events

8.1.8 Month 12/ (Visit 8, Day 365)

- Collect concomitant medications and treatments
- Focused knee assessment
- Perform vital signs
- MRI
- PROs
- Assess adverse events
- Immunogenicity testing (Arm 3 only)

8.1.9 Unscheduled Visits

Unscheduled visits are allowed as needed and at the discretion of the treating investigator for more frequent follow-up than what is required in the protocol. The treating investigator will determine the appropriate assessments to be conducted during this visit based on the reason for the Unscheduled Visit. Data from the Unscheduled visits will be captured in the EDC.

8.1.10 End of Study Visit

If for any reason the study is discontinued early (according to section 7.5), all subjects should complete the End of Study visit and be followed for 90 days for any SAEs that are ongoing at the End of Study visit. All AEs potentially related to study participation will be followed until resolution prior to study completion. Open AEs that are unrelated to the study participation will not be followed beyond subject's study completion. Assessments completed during this visit will be at the discretion of the treating investigator and what is best for the safety of the subjects. Data from end of study visit will be captured in the EDC.

8.2 Clinical Assessments and Procedures

8.2.1 Informed consent and HIPAA Authorization

All subjects must agree to participate by signing and dating the informed consent and HIPAA Authorization prior to any study specific screening procedures being performed.

Consent must be documented by the subject's dated signature on a consent and HIPAA form along with the dated signature of the person conducting the consent discussion.

A copy of the signed and dated consent form and HIPAA Authorization should be given to the subject before participation in the trial. The original documents should be filed in the subject's study chart.

The initial informed consent form, any subsequent revised written informed consent form and any written information provided to the subject must receive the IRB's approval/favorable opinion in advance of use. The subject should be informed in a timely manner if new information becomes available that may be relevant to the subject's willingness to continue participation in the trial. The informed consent will adhere to IRB requirements, applicable laws, and regulations.

8.2.2 Assignment of Screening Number

Once informed consent and HIPAA Authorization have been documented, the site will assign the subject a screening number. Each site will be responsible for maintaining their Screening Log of consented subjects.

8.2.3 Inclusion/Exclusion Criteria

All inclusion and exclusion criteria will be reviewed by the investigator or qualified designee to ensure that the subject qualifies for the study.

8.2.4 Demographics

Subject demographics will be obtained by the investigator or qualified designee.

8.2.5 Medical and Surgical History

A medical and surgical history will be obtained by the investigator or qualified designee. Medical history will include all active conditions, and any condition deemed clinically relevant by the treating investigator. Surgical history will include any past surgeries deemed clinically relevant by the treating investigator.

8.2.6 Prior Medications

The investigator or qualified designee will review prior medication use and record any medication taken by the subject within 21 days before signing the informed consent.

8.2.7 Concomitant Medications and Treatments

The investigator or qualified designee will record any pain medication (including anti-inflammatories, NSAIDS, etc.), knee injections and surgeries the subject required during the study until the subject's final study visit.

8.2.8 Physical Examination

Focused knee assessment will be performed. Please refer to the Operations Manual to ensure all required assessments are performed during the exam. At the 7-day follow-up and 30-day follow-up visits, the assessment will also include observation of the knee injection site for all subjects and observation of the harvest site for subjects in Arm 1 and Arm 2.

8.2.9 Vital Signs

The investigator or qualified designee will obtain vital signs. Please refer to the Operations Manual for details.

8.2.10 MRI

MRIs will be performed using cartilage mapping sequences for each location based on their machines. The baseline MRI scans and each subsequent MRI scan must be performed on the same machine with the same sequence. Further details are described in the MRI protocol.

8.2.11 Patient Reported Outcomes

8.2.11.1 Visual Analog Pain Scale (VAS)

8.2.11.2 Euro-Quality of Life (EQ5D 3L)

8.2.11.3 Knee Injury and Osteoarthritis Outcome Score (KOOS)

8.2.11.4 Patient-Reported Outcomes Measurement Information System (PROMIS 29)

8.2.12 Adverse Event Monitoring

The investigator or qualified designee will assess each subject to evaluate for potential new or worsening AEs at each study visit and more frequently if clinically indicated. Adverse experiences will be graded and recorded throughout the study according to CTCAE (Common Terminology Criteria for Adverse Events). Toxicities will be characterized in terms regarding seriousness, causality/attribution, toxicity grading, and action taken.

Subjects must agree to notify the investigator or study team member as soon as possible of any failure of proper use of your birth control method, or if a subject becomes pregnant, either of which may result in being withdrawn from the study or having limited participation in the study. Subjects who become pregnant while on the study will be followed for AEs/SAEs that may occur for up to 30 days following the birth of the child.

8.2.13 Physical Therapy

Subjects will follow an at home physical therapy program, see Appendix 12.1, unless otherwise directed by the investigator. Instructions for the at home physical therapy will be provided to the subject, by the study team, at the treatment visit.

The study team will provide subjects with a physical therapy log to track compliance with the recommended daily activity (according to Appendix 12.1). Subjects will be required to return the log at the 7-day and 30-day follow-up visits. The study team will review the log for compliance.

8.2.14 Cell Characterization

Cell characterization that will be performed includes:

- Cell viability assessment including apoptosis assay
- Cell phenotype assessment via comprehensive flow cytometry or mass cytometry
- Single-cell transcriptome analysis
- Secretome assessment from both initial aspirate and rescue culture supernatant

*Specimen processing, storage and shipment details can be found in the Laboratory Manual.

8.2.15 Local Laboratory Assessments

Laboratory assessments will be collected for subject safety.

8.2.15.1 Hematology

Hematology can be evaluated in either the fed or fasted state and consist of a complete blood count with differential count performed as needed at the investigator’s discretion.

8.2.15.2 Clinical Chemistry

A comprehensive metabolic panel OR a basic metabolic panel and liver function panel will be evaluated in the fed or fasted state Additional clinical chemistry testing will be performed as needed at the investigator’s discretion. The subject’s ALT and total bilirubin must be evaluated in order to determine study eligibility.

8.2.15.3 Coagulation Assays

Coagulation assays will be evaluated in the fed or fasted state and consist of prothrombin time (PT; sec) or international normalized ratio (INR). Activated partial thromboplastin time (APTT; sec) may be ordered at the discretion of the treating investigator.

8.2.15.4 Immunogenicity Testing

Immunogenicity Testing can be evaluated in either the fed or fasted state and consist of a panel reactive antibodies (PRA) on the recipient of the umbilical cord tissue at 30 days and at study completion. All subjects in Arm 3 will have a blood draw to maintain blinding.

8.2.16 Pregnancy Testing

Pregnancy testing for human chorionic gonadotrophin (hCG) will be performed on female subjects of childbearing potential (refer to eligibility criteria). This may be done using a urine or serum sample. Pregnancy testing will be done at two time points:

a) **Screening Visit**- if the test is positive the subject will be a screen failure.
b) **Treatment Visit** (prior to procedure/knee injection)- if the test is positive, within 7 days of the Treatment Visit, the subject will be discontinued from the study.

8.3 Protocol Deviations

Assessments not performed at a required visit per protocol should be documented as a deviation from the protocol, regardless of the reason. When a protocol deviation occurs it should be discussed with the treating investigator and documented on the Protocol Deviation Form detailing the deviation. Protocol Deviation forms will be signed by the treating investigator and reviewed by the sponsor's representative.

8.4 Adverse Event Reporting and Safety Monitoring

8.4.1 Adverse Events

An adverse event is defined as any untoward or unfavorable medical occurrence in human subject, including any abnormal sign, symptom, or disease, temporally associated with the subject's participation in the research, whether or not considered related to the subject's participation in the research.

The investigator or qualified designee will assess each subject to evaluate for potential new or worsening AEs as specified in the SoA and more frequently if clinically indicated. Adverse experiences will be graded and recorded throughout the study according to CTCAE.

8.4.2 Serious Adverse Events

If the adverse event meets any of the criteria below, it is regarded as a serious adverse event (SAE):

a) Led to death
b) Led to serious deterioration in the health of the subject, that either resulted in
    a. A life-threatening illness or injury, or

b. A permanent impairment of a body structure or a body function, or
c. In-patient or prolonged hospitalization, or
d. Medical or surgical intervention to prevent life-threatening illness or injury or permanent impairment to a body structure or a body function

8.4.3 AE and SAE Collection

All AEs and SAEs will be collected from the signing of the informed consent form until the one-year follow-up visit at the time points specified in the SoA (Section 1.3) as required by applicable regulation and best practice.

All SAEs will be recorded and reported to the sponsor and the pharmacovigilance team at Advanced Clinical within 24 hours of the study personnel becoming aware of the event. Please utilize the approved SAE data collection forms provided by Advanced Clinical to submit the notifications and updates. The investigator will submit any updated SAE data to the sponsor within 24 hours of it being available.

Investigators are not obligated to actively seek AE or SAE after conclusion of the study participation. However, if the investigator learns of any SAE, including a death, at any time after a subject has been discharged from the study up to a 30 day follow-up period, and he/she considers the event to be reasonably related to the study intervention or study participation, the investigator must promptly notify the sponsor.

8.4.4 Follow-up of AEs and SAEs

After the initial AE/SAE report, the investigator is required to proactively follow each subject at subsequent visits/contacts. All AE/SAEs, will be followed until resolution, stabilization, the event is otherwise explained, or the subject is lost to follow-up (Lost to follow-up is defined in Section **Error! Reference source not found.** of the protocol).

8.4.5 Adverse Event and Serious Adverse Event Reporting

Adverse events and serious adverse events will be reported to the FDA, the Sponsor and IRB in accordance to standard policy. These will be listed per site and reviewed by the Site PI, Contract Research Organization (CRO), study monitors, Sponsor's Medical Monitor and the Data Safety Monitoring Board (DSMB).

8.4.6 Investigational New Drug (IND) Safety Reports

Written Investigational New Drug (IND) safety reports will be submitted to the FDA by the IND sponsor, for serious unexpected adverse reactions within 15 calendar days of learning of its occurrence. If the event is fatal or is deemed to be life threatening, the report will be made within 7 calendar days.

8.4.7 Unanticipated Problems (Emory IRB Requirement)

The IND sponsor will make an assessment of whether an adverse event constitutes an unanticipated problem (UP) posing risks to subjects or others, per the Emory IRB Reportable Events guidelines. The IND Sponsor's assessment will be provided to the Emory IRB, which in turn will make a final determination. If the Emory IRB determines the event is an UP it will notify the appropriate regulatory agencies and institutional officials. Reporting of the Emory IRB's final determination should be submitted to the site's IRB of record per their policies and guidelines. See MILES Study Operations Manual for further details regarding reporting process.

9.0 Statistical Considerations

9.1 Statistical Hypothesis

An experienced statistician trained in multicenter clinical trials has provided statistical justification. De-identified early pilot data, outside of this study, from the Emory Department of Orthopedics provided sample data for estimation.

Rigorous statistical analyses were performed based on the preliminary data with autologous MSC cell injections and data in the literature to design a randomized controlled study that will provide sufficient power to compare data from four different sites. The collaborative work will involve recruitment sites with an established track record in using stem cells for the treatment of knee osteoarthritis. In addition, these sites have a large clinical volume to complete the study as proposed.

While 120 subjects per site would provide sufficient power to achieve statistical differences, this number reflects a conservative estimate compared to the number of subjects that each site recruits for stem cell injections per year based on retrospective evaluations. The sample size of 120 subjects amongst 4 sites for each of the four arms is substantially greater than the other less rigorously controlled trials and the number also accounts for reasonably unlikely yet unforeseen variability between recruitments.

9.2 Sample Size Determination

The total estimated sample size for the proposed intention-to treat, parallel-group, multicenter, randomized, controlled trial is 480 subjects. The primary endpoints are Visual Analog Scale (VAS) pain score and the pain subsection of the Knee Injury and Osteoarthritis Outcomes (KOOS). The sample size calculations are based on improvements (from baseline to 1-year) in the visual analog scale (VAS) pain score and total KOOS. Total KOOS was used as a proxy and should ensure that the study is amply powered.

Assuming a decline of 1 point on average in pain in the Control arm (corticosteroids) and a decline on average of 2.5 points in a treatment arm (mesenchymal stem cells) and an estimated standard deviation on change of 3.5, the proposed sample sizes (n=121 subjects

per group or 484 total subjects) will provide 91% power to detect a difference on change of 1.5 points at the two-sided 5% significance level if the true difference between treatment groups is 1.5 points (two-sided two-sample equal variance t-test).

Assuming an increase of 10 points on average in total KOOS in the Control arm (corticosteroids) and an increase on average of 20 points in a treatment arm (mesenchymal stem cells) and an estimated standard deviation on change of 20, the proposed sample sizes (n=121 subjects per group or 484 total subjects) will provide 97% power to detect a difference on change of 10 points at the two-sided 5% significance level if the true difference between treatment groups is 10 points (two-sided two-sample equal variance t-test).

Each of the four participating sites will accrue 30 subjects to each of the 4 treatment groups. Cohort retention is expected to be 90% at 1-year.

9.3 Efficacy Analyses

The statistical analysis plan (SAP) has been developed and describes the subject populations to be included in the analyses, and procedures for accounting for missing, unused, and spurious data. This section is a summary of the planned statistical analyses of the primary and secondary endpoints.

The primary analysis of the data will be performed according to subjects' original treatment assignment (i.e., intention-to-treat analyses) regardless of their compliance to treatment and the inclusion of all data from all subjects randomized in the final analysis.

9.3.1 Efficacy Variables

Primary efficacy variables:

- Visual Analog Scale (VAS) pain score
- KOOS Pain Score

Secondary efficacy variables:

- Total KOOS Score
- KOOS Symptoms Score
- KOOS ADL Score
- KOOS Sports/Recreation Score
- KOOS Quality of Life (QoL) Score
- European Quality of life Score (EQ5D 3L) Index

9.3.2 Primary and Secondary Efficacy Analyses

Eight separate repeated-measures analysis will be performed on the eight efficacy variables described above. Each of the eight variables will be used as a dependent variable. The following model building method will be used for each analysis. Using Statistical Analysis System (SAS) MIXED Procedure (version 9.4; SAS Institute, Cary, NC), a linear mixed model will be fit on each efficacy variable. The model will include site, treatment arm, time on study (baseline and 1, 3, 6, 9

and 12 months on study), and treatment arm by time interaction as fixed effects. Subject ID will be the random effect. A compound-symmetric variance-covariance form in repeated measurements will be assumed and robust estimates of the standard errors of parameters will be used to perform statistical tests and construct 95% confidence intervals. The model-based means are unbiased with unbalanced and missing data, assuming missing data are non-informative. A P-value ≤0.05 will be considered statistically significant for all the terms in the mixed model. The statistical test for interaction between time on study and treatment will be the overall hypothesis test to determine whether efficacy variables in the four treatment arms changed significantly during 12 months of follow-up If mean scores in the treatment arms are consistently different or similar over time (i.e., no statistical interaction) then the main effect test for treatment will be used as the primary hypothesis test to compare the treatment arms. The primary study results from this model will be the mean score and 95% confidence interval for each of the 4 treatment arms and the treatment mean differences and 95% confidence intervals.

Assuming a significant treatment effect on efficacy score change is identified (i.e., the interaction test is significant at P = 0.05) then the six pairwise treatment comparisons on pain score change will be performed. A Bonferroni correction (p=0.05/6=0.0083) for multiple comparisons will be applied. Specific statistical tests on efficacy score change will be done within the framework of the mixed effects linear model. All statistical tests will be 2-sided and adjusted for multiple comparisons. The same Bonferroni method will be used if the statistical interaction between treatment group and time on study is not significant but the main effect test for treatment is significant.

Missing values will be excluded from the repeated measures analyses. Unknown values for ethnicity and gender will be included as a separate unknown category in the models if they represent more than 5% of the subjects.

9.3.3 Efficacy Analyses across Subgroups

The heterogeneity of treatment effects across age, sex, and ethnicity will be evaluated using a statistical test for interaction terms. Specifically, effects of treatment in subgroups will be determined by including the additional interaction terms between treatment and the three subgroups (age, sex, and ethnicity) in the repeated measures model described above for the eight efficacy variables.

The following variables will be treated as categorical variables in all of the linear mixed models.

- Treatment arms: CSI (reference), BMAC, ADP, UCT
- Age: ≤65 (reference) vs. >65
- Sex: female vs male (reference)

- Ethnicity: Hispanic vs non-Hispanic
- Time: 0, 1, 2, 3, 4, 5 representing baseline and 1, 3, 6, 9 and 12 months follow-up visits

9.4 Safety Analyses

9.4.1 Trial Drug Exposure

Descriptive statistics will be summarized for cell count, cell viability, endotoxin completion status, and 14-day sterility testing status by BMAC, UCT and SVF treatment arms. The treatment data for BMAC, UCT, SVF and CSI arms will be provided in four separate data listings. Cell count and cell viability data will also be included in the listings for BMAC, UCT and SVF treatment arms.

9.4.2 Adverse Events

All adverse events (AEs) and serious adverse events (SAEs) will be classified by System Organ Class (SOC) and Preferred Term (PT) according to the Medical Dictionary of Regulatory Activities (MedDRA) version 21.1 for the safety population. The number and percentage of subjects reporting AEs, as well as the number of events, in each treatment group will be tabulated by SOC and PT; by SOC, PT, and maximum severity; and in relationship to study treatment, SOC and PT. If a subject has multiple occurrences of the same SOC and PT, then the subject will be counted only once for the SOC and PT using the most sever occurrence for the summarization by maximum severity. Presentation by SOC and PT will display SOC sorted alphabetically and PT by descending frequency within SOC.

9.4.3 Continuous Monitoring of Specific Adverse Events

In addition, the incidence of specific safety related events of concern will be continuously monitored separately in each treatment arm at each center throughout the study to determine if any of their observed subject-based incidence rates exceed a threshold incidence rate of concern pre-specified for each particular event (for example infection and allergic reaction related to study harvest procedure and/or knee injection).

These rules will be implemented by Advanced Clinical continuously monitoring the occurrence of any events to determine if the currently observed rate of each exceeds the corresponding threshold rate of concern with a pre-specified level of confidence. The stopping rule will be considered to be met if the lower 95% confidence limit on the observed incidence rate exceeds the corresponding threshold level of concern for that event. If any stopping rule is met, enrollment and randomization will be suspended pending expedited DSMB review.

The following table describes the minimum number of events out of the selected numbers of subjects randomized to each treatment arm at each site

which, if equaled or exceeded, would satisfy the stopping rule for each event of concern.

Table 1. Study Termination Rules for Infection and Allergic Reaction:

Minimum Number of Subjects with Events Meeting Stopping Rule for Infection and Allergic Reaction (infections and/or allergic reactions must be related to the harvesting procedure and/or knee injection) for Selected Numbers of Subjects Randomized Per Treatment Arm at each Center Using a Threshold Incidence Rate of Concern of 10%

Number of Subjects with Event	Number of Randomized Subjects Total	Incidence Rate	Lower 95% Confidence Limit, Wilson score method
1	5	20%	4%
**2**	**5**	**40%**	**12%**
1	10	10%	2%
2	10	20%	6%
**3**	**10**	**30%**	**11%**
**4**	**10**	**40%**	**17%**
4	20	20%	8%
**5**	**20**	**25%**	**11%**
**6**	**20**	**30%**	**15%**
5	30	17%	7%
**6**	**30**	**20%**	**10%**
**7**	**30**	**23%**	**12%**

Examples above illustrate threshold number of events that exceed the stopping rule (highlighted in bold); non-highlighted entries illustrate the observed incidence rate and the lower 95% confidence limit that does not exceed the stopping rule threshold of 10%

For each treatment arm at each center: if the lower confidence interval for the estimated infection and allergic reaction rate is 10% or higher, then enrollment and randomization will be suspended pending DSMB review. Infection and/or allergic reactions must be related to the harvesting procedure and/or knee injection.

9.4.4 Analysis of Adverse Events

Additionally, the following AE-related specific analysis will be performed.

1. Frequency and percentages of each event will be summarized by treatment arm for the following:

- AEs
- AE in relationship to study treatment (not-related, possibly related, or related)
- AE Severity
- SAEs
- SAE in relationship to study treatment (not-related, possibly related or related)
- SAE Severity
- Any Laboratory Toxicities

2. Allergic reaction and infection will be summarized as one category and corresponding number and proportions will be provided for each CTCAE grade level by treatment arm. These AEs will be counted only once per subject as the most severe level (i.e., maximum severity) reported across the six study assessments/visits during the 1-year follow-up period and compared between treatment arms with a chi-square or Fisher's exact test. The 95% confidence intervals will also be provided for the proportions.
3. Estimates of infection at harvest site or treated/targeted knee by treatment arms and time on study will be estimated and compared by performing a generalized estimating equations (GEE) analysis of the repeated binary responses within participant. The subjects will be clustered within center. This analysis will be implemented via the SAS GENMOD Procedure, using an exchangeable correlation structure for the repeated data within subjects (binomial-logit model). Infection rates and 95% confidence intervals will be used to summarize the data by treatment group and clinical visit.
4. Subjects who become pregnant while on the study will be followed for AEs/SAEs that may occur for up to 30 days following the birth of the child.

9.5 Interim Analyses and Data Monitoring

No formal interim analyses for efficacy will be performed for this study. A Data Safety and Monitoring Committee (DSMB) will review safety data bi-annually throughout the course of this study with one possible additional meeting within the second 6-month period. The DSMB objectives and operational details are detailed in the DSMB charter.

10.0 Ethical Considerations and Compliance with Good Clinical Practice

10.1 Statement of Compliance

This study will be conducted in accordance with this protocol, the International Conference on Harmonization (ICH) GCP, 21 Code of Federal Regulations (CFR), and applicable national and local regulatory requirements.

Before enrollment of subjects into this study, the IRB will review and approve/give favorable opinion on the protocol, ICF, any promotional material/advertisements, and any other written information to be provided to the subjects. The IB/IFU will be provided to

the IRB for review. The IRB's composition or a statement that the IRB's composition meets applicable regulatory criteria will be documented.

If the protocol or any other information given to the subject is amended, the revised documents will be reviewed and approved/given favorable opinion by the IRB, where applicable. The protocol amendment will only be implemented upon the sponsor's receipt of approval and, if required, upon the sponsor's notification of applicable regulatory authority(ies). Amendments to the protocol that eliminate an apparent immediate hazard to subjects do not require pre-approval by the IRB.

10.2 Participating Centers

Participating clinical sites must have an appropriate IRB governance since they are actively engaged in research and provide informed consent. The protocol and consent forms will be approved for the sponsor by a central IRB review prior to release to participating sites. Health Insurance Portability and Accountability Act (HIPAA) and applicable local regulations will be followed by each participating institution in accordance with each institution's requirements. The participating sites will obtain approval from their corresponding review boards in accordance with their local procedures and institutional requirements.

The investigator is required to keep accurate records to ensure the conduct of the study is fully documented.

The investigational sites participating in this study will maintain the highest degree of confidentiality permitted for the clinical and research information obtained from subjects participating in this study. Unless required by the laws permitting copying of records, only the coded identity associated with documents or other subject data may be copied (obscuring any personally identifying information). Authorized representatives, as noted above, are bound to maintain the strict confidentiality of medical and research information that may be linked to identify individuals. The investigational site will normally be notified in advance of auditing visits.

10.3 Informed Consent

Written informed consent will embody the elements of informed consent as described in the Declaration of Helsinki and the ICH Guidelines for GCP and will be in accordance with all applicable laws and regulations. Investigators will enroll subjects according to the study eligibility criteria and in compliance with 21 CFR 50. The investigator will exercise no selectivity so that no bias is introduced from this source.

10.4 Study Subject Confidentiality

The investigator will comply with applicable subject privacy regulations/guidance as described as per HIPAA and any additional local regulations.

The investigational sites participating in this study will maintain the highest degree of confidentiality permitted for the clinical and research information obtained from subjects. Medical and research records will be maintained at each site in the strictest confidence, however, as a part of the quality assurance and legal responsibilities of an investigation, the investigational site must permit authorized representatives of the sponsor(s) and regulatory agencies to examine (and when required by applicable law, to copy) records for the purposes of quality assurance reviews, audits and evaluation of the study safety and progress. Unless required by the laws permitting copying of records, only the coded identity associated with documents or other subject data may be copied (obscuring any personally identifying information). Authorized representatives, as noted above, are bound to maintain the strict confidentiality of medical and research information that may be linked to identify individuals. Study records with the study subject's information for internal use at the clinical sites will be secured at the study site during the study. At the end of the study, all records will continue to be kept in a secure location for at least 2 years post market approval in the applicable region. There are no plans to destroy the records

10.5 Study Documentation and Case Report Forms (CRFs)

The data collection tool for this study will be sponsor defined eCRFs to be completed by study-site personnel. The investigator will maintain complete and accurate study documentation in a separate file. Study documentation may include medical records, records detailing the progress of the study for each subject, signed informed consent forms, drug disposition records, correspondence with the Institutional Review Board (IRB) and the study monitor/sponsor, screening and consent information, CRFs, SAE reports, laboratory reports, subject diaries, data clarifications requested by the sponsor or designee, and any other documentation deemed relevant and pertinent to the study and the study subjects. Subject data necessary for analysis and reporting will be entered into a validated database or data system in accordance with Title 21 of the Code of Federal Regulations (21 CFR) Part 11. Clinical data management will be performed by Advanced Clinical in accordance with applicable data management standards and data cleaning procedures. The investigator is responsible for the procurement of data and for the quality of data recorded on the eCRFs. The eCRF will be electronically signed by the investigator listed on Form FDA 1572.

The handling of data by the sponsor and the data management vendor (Advanced Clinical), including data quality assurance, will comply with regulatory guidelines and the standard operating procedures of the sponsor or designee. Data management and control processes specific to the study will be described in the Data Management Plan as created for this study by Advanced Clinical CRO.

10.6 Monitoring of the Study

Advanced Clinical will be responsible for data monitoring, data integrity, and data analysis. The study will be routinely monitored to ensure compliance with the study protocol and the overall quality of data collected. Throughout the course of the study, the

study monitor will make frequent contacts with the investigator through telephone calls and on-site visits. During the on-site visits, source documentation and the eCRFs will be reviewed for completeness and adherence to the protocol. As part of the data audit, source documents will be made available for review by the study monitor. The study monitor may periodically request review of the investigator study file to assure the completeness of documentation in all respects of clinical study conduct.

The study monitor will verify that each subject has proper consent documentation from the subject for study procedures and for the release of medical records to the sponsor, FDA, other regulatory authorities, and the IRB. The investigator or appointed delegate will receive the study monitor during these on-site visits and will cooperate in providing the documents for inspection and respond to inquiries. In addition, the investigator will permit inspection of the study files by authorized representatives of the regulatory agencies.

Upon completion of the study, the study monitor will arrange for a close out visit, at each participating site, as defined in the Clinical Monitoring Plan (CMP). Study files should be secured for the appropriate time period.

10.7 Direct Access to Source Data/Documents

The investigator/study site will cooperate and provide direct access to study documents and data, including source documentation for monitoring by the study monitor, audits by the sponsor or sponsor's representatives, review by the Ethics Committee, and inspections by applicable regulatory authorities. If contacted by an applicable regulatory authority, the investigator will notify the sponsor of contact, cooperate with the authority, provide the sponsor with copies of all documents received from the authority, and allow the sponsor to comment on any responses.

10.8 Medical Monitor and Data Safety Monitoring Board (DSMB)

All adverse events will be recorded on the adverse event forms, and the treatment related SAEs will be sent to the IRB, per their reporting requirements, and to sponsor. The study medical monitor or designee will review all adverse event reports. Further details are captured in the study medical monitoring plan, data management plan, and safety management plan created for this study by Advanced Clinical and maintained in the study Trial Master File.

The DSMB is responsible for monitoring the safety of the trial subjects, and data integrity during periodic and ad hoc reviews. The DSMB will meet every 6 months; it can recommend to the sponsor and the study executive committee the continuation of study as planned, modification of the protocol, or the stopping of the study.

The sponsor will ensure the timely communication of the safety and efficacy data to the DSMB according to the agreed upon schedules or within reasonable time if requested for

an ad hoc analysis. The DSMB will meet bi-annually and ad hoc as specified in its charter.

Data accrual will be performed continuously as the study is being performed. Advanced Clinical, LLC, is the CRO that is responsible for the data management plan and integrity of the study data. The study medical monitor will be working continuously with Advanced Clinical to assess the data in regard to safety concerns and will recommend ad hoc review by the DSMB as necessary.

10.9 Publication Policy

Manuscript submission for publication of the results of this study, or any aspect of work directly related to the study, will be decided by the investigators. Any investigator can submit a publication concept proposal including proposed authorship order to the rest of the investigators for review, which will take place within 30 days after a proposal has been submitted.

10.10 Maintenance and Retention of Records

The investigator will retain study documentation and data (electronic case report forms) in accordance with applicable regulatory requirements, institutional policy and the document and data retention policy, as described in the Data Management Plan or other supporting documents as applicable.

Signed consent forms for each subject will be coded with the subject's unique study identification number and stored locally at each clinical site.

11.0 References

1. Kim, Jae-Do, et al. "Clinical outcome of autologous bone marrow aspirates concentrate (BMAC) injection in degenerative arthritis of the knee." *European Journal of Orthopaedic Surgery & Traumatology* 24.8 (2014): 1505-1511.

2. Jo, Chris Hyunchul, et al. "Intra-articular Injection of Mesenchymal Stem Cells for the Treatment of Osteoarthritis of the Knee: A 2-Year Follow-up Study." *The American journal of sports medicine* 45.12 (2017): 2774-2783.

3. Shapiro, Shane A., et al. "A prospective, single-blind, placebo-controlled trial of bone marrow aspirate concentrate for knee osteoarthritis." *The American journal of sports medicine* 45.1 (2017): 82-90.

4. Buckwalter, Joseph A., et al. “The Impact of Osteoarthritis: Implications for Research.” Clinical Orthopaedics and Related Research (1976-2007): October 2004-Volume 427-Issue-pp S6-S15

5. Micromedix: Bone Marrow Harvesting-General Information. Accessed through Sanford Health CareNotes August 2, 2018

6. Cheng, Jiango, M.D., Ph.D. and Abdi, Salahadin, M.D., PhD. “Complications of Joint, Tendon, and Muscle Injections.” *Tech Reg Anesth Pain Manag.* 2007 Jul; 11(3): 141-147

7. Micromedix: Magenetic Resonance Imaging-General Information. Accessed through Sanford Health CareNotes August 2, 2018

8. Illoouz, Yves-Gerard., and Aris Sterodimas, eds. Adipose Stem Cells and Regenerative Medicine. New York: Springer Heidelberg Dordrecht, 2011. Print

12.0 Appendices

12.1 Joint Rehab

Establishing any home physical therapy program requires incorporating anticipated physiological changes with expected joint dysfunction. The following is a guideline to help you with your home physical therapy program following biologic injections to weight bearing joints. These guidelines were developed from research findings, therapist and patient feedback and clinical experience from physicians who routinely utilize stem cells.

Post Procedure Day	Precautions	Home Program
**1-7**	• WBAT (Weight Bearing As Tolerated), but PWB (Partial Weight Bearing) with crutches is allowed for patients with moderate to severe pain<br>• Avoid any heavy loading activities such as hiking, running, or heavy weight lifting	• Bracing if indicated by physician<br>• Gentle ROM (range of motion) if pain is not severe
**8-14**	• WBAT<br>• Avoid impact activities<br>• Avoid heavy weight lifting to affected joint	• Follow the **Knee Range of Motion** and **Strengthening** Post-Procedure **Days 8-14** instructions provided to you by the study team<br>• May start swimming and biking (low resistance)
**15-28**	• Avoid impact activities	• Follow the **Knee Range of Motion** and **Strengthening** Post-Procedure **Days 15-28** instructions provided to you by the study team<br>• Can increase biking/ swimming activities<br>• May resume light aerobic activities such as walking<br>• Weight lifting/ strength training as tolerated

12.2 Lab Value Reference Ranges

Please refer to your site’s local laboratory reference ranges.
