# Supplementary material for: Cell-based versus corticosteroid injections for knee pain in osteoarthritis: a randomized phase 3 trial
Source: Nat Med. 2023 Nov 2;29(12):3120–6. doi: 10.1038/s41591-023-02632-w (PMC10719084; doi:10.1038/s41591-023-02632-w)
Supplement: Supplementary file 2 — Reporting Summary [file 41591_2023_2632_MOESM2_ESM.pdf]

nature portfolio

Corresponding author(s): Ken Mautner, MD

Last updated by author(s): May 11, 2023

Reporting Summary

Nature Portfolio wishes to improve the reproducibility of the work that we publish. This form provides structure for consistency and transparency in reporting. For further information on Nature Portfolio policies, see our Editorial Policies and the Editorial Policy Checklist.

Statistics

For all statistical analyses, confirm that the following items are present in the figure legend, table legend, main text, or Methods section.

n/a	Confirmed	
☐	☒	The exact sample size (*n*) for each experimental group/condition, given as a discrete number and unit of measurement
☐	☒	A statement on whether measurements were taken from distinct samples or whether the same sample was measured repeatedly
☐	☒	The statistical test(s) used AND whether they are one- or two-sided<br>*Only common tests should be described solely by name; describe more complex techniques in the Methods section.*
☐	☒	A description of all covariates tested
☐	☒	A description of any assumptions or corrections, such as tests of normality and adjustment for multiple comparisons
☐	☒	A full description of the statistical parameters including central tendency (e.g. means) or other basic estimates (e.g. regression coefficient) AND variation (e.g. standard deviation) or associated estimates of uncertainty (e.g. confidence intervals)
☐	☒	For null hypothesis testing, the test statistic (e.g. *F*, *t*, *r*) with confidence intervals, effect sizes, degrees of freedom and *P* value noted<br>*Give P values as exact values whenever suitable.*
☒	☐	For Bayesian analysis, information on the choice of priors and Markov chain Monte Carlo settings
☐	☒	For hierarchical and complex designs, identification of the appropriate level for tests and full reporting of outcomes
☒	☐	Estimates of effect sizes (e.g. Cohen's *d*, Pearson's *r*), indicating how they were calculated

Our web collection on statistics for biologists contains articles on many of the points above.

Software and code

Policy information about availability of computer code

Data collection	For data collection, Medidata Rave 2022.1.0 was the version in use at the time of the database lock. We used Rave Coder 2022.1.0 as the tool for medical coding, Rave Medical Imaging v2021.3.3 to collect the MRI data and Rave RTSM 2022.2.0 for randomization.
Data analysis	Statistical analysis will be performed using SAS® (version 9.4).<br>Handling of Missing Data<br>For primary analyses, missing data for VAS pain scores (over 1 Month) and KOOS pain scores will be imputed using Multiple Imputation (MI) under the Missing At Random (MAR) assumption. The following steps will be followed:<br><br>1. For VAS pain scores (over 1 Month) and KOOS pain scores, the missingness pattern in the data will be evaluated. If the pattern is not monotone, the MCMC method of SAS PROC MI will be used to make it monotone. The single chain method will be used, with 200 burn-in iterations and 100 iterations between imputations. The minimum values for imputed variables will be set to 0, in order to force PROC MI to redraw another value for imputation when an intended imputed value is less than the 0. For VAS pain scores (over 1 Month) and KOOS pain scores, the maximum value for imputed variables will be set to 100, in order to force PROC MI to redraw another value for imputation when an intended imputed value is greater than the 100. For VAS pain scores (over 1 Month) only, imputed values will be rounded to the nearest integer. The seed number will be set to 202394 and fifty (50) imputations will be created.<br><br>2. SAS PROC MI will be used for imputing missing values of data with monotone missing pattern. If the MCMC method of step 1 was previously employed, one imputation will be made using each of the fifty (50) MCMC-imputed datasets. If the MCMC method of step 1 was not previously employed, fifty (50) imputations will be created assuming the data are Missing At Random. The seed number will be set to 202394.

These imputations will use the following model:

For VAS pain scores (over 1 Month) and KOOS pain scores, a linear regression model will be used with covariates for treatment and non-missing VAS pain score (over 1 Month) and KOOS pain score from earlier scheduled time points including baseline.

3. The imputed datasets will be analyzed as specified in the primary efficacy analyses section.

4. The resulting analysis on the imputed datasets will then be combined to produce a single set of statistics as follows:

For VAS pain scores (over 1 Month) and KOOS pain scores, results from the MMRM analysis will be combined using the SAS PROC MIANALYZE.

Primary Efficacy Analyses
Using SAS Proc Mixed procedure, a mixed model will be fit on each efficacy variable. The models will include site, treatment arm, time on study (baseline and 1,3,6,9, and 12 months on study), and treatment arm by time interaction as fixed effects. A compound-symmetric variance-covariance form in repeated measurements will be assumed and robust estimates of the standard errors of parameters will be used to perform statistical tests and construct 95% confidence intervals

Analysis of MRI data
Absolute change in MRI Score from baseline to month 12 will be analyzed on MRI endpoints using an ANCOVA with site, treatment arm and baseline MRI Score as fixed effects; the p-values for the treatment comparison, estimates of the treatment difference and the 95% confidence interval of the difference will be generated from the ANCOVA model. SAS Proc Mixed will be used to analyze MRI data.

For manuscripts utilizing custom algorithms or software that are central to the research but not yet described in published literature, software must be made available to editors and reviewers. We strongly encourage code deposition in a community repository (e.g. GitHub). See the Nature Portfolio guidelines for submitting code & software for further information.

Data

Policy information about availability of data

All manuscripts must include a data availability statement. This statement should provide the following information, where applicable:

- Accession codes, unique identifiers, or web links for publicly available datasets
- A description of any restrictions on data availability
- For clinical datasets or third party data, please ensure that the statement adheres to our policy

Provide your data availability statement here.

Human research participants

Policy information about studies involving human research participants and Sex and Gender in Research.

Reporting on sex and gender	This study included both males and females that met the inclusion/exclusion criteria. Sex of subject was self reported with no data collected regarding gender preference. There was a significant interaction between treatment group sex (P=0.01) for VAS pain score over 1 Month.
Population characteristics	ANALYSIS POPULATIONS<br>5.1 Intent-to-treat Population<br>The ITT population is defined as all subjects who signed the informed consent and were randomized. The ITT population will be used for all efficacy analysis as a primary analysis set with treatment assignment based on randomization.<br>5.2 Safety Population<br>The Safety population is defined as all subjects who have received study treatment. The safety population will be used for all safety analyses with treatment actually received.<br>5.3 Per Protocol Population<br>The Per-Protocol Population (PP) includes subjects in the ITT population without any major protocol deviations. A major protocol deviation is a deviation that may significantly impact the completeness, accuracy, and/or reliability of the trial data; that may significantly affect a subject's rights, safety, or well-being (ICH E3 R1 Guidelines 2013).<br>At the primary efficacy analysis, protocol violators resulting in exclusion from the PP population will be identified by the sponsor and documented prior to the database freeze.
Recruitment	Eligible subjects were recruited at five participating sites primarily from the patients already being seen for knee osteoarthritis in those clinics. Subjects also contacted the participating sites from contact information provided in the clinicaltrials.gov study description, from IRB approved recruitment materials, and by word of mouth from subjects already in the study. There is no potential for self-selection bias or other biases that would impact the results.
Ethics oversight	The study protocol was approved by Western Institutional Review Board (WIRB), as well as by local site institutional IRBs as required by those sites. Emory University required local IRB approval due to the sponsor to the FDA being a faculty member, Dr. Scott Boden. Duke University required local IRB approval as well.

Note that full information on the approval of the study protocol must also be provided in the manuscript.

Field-specific reporting

Please select the one below that is the best fit for your research. If you are not sure, read the appropriate sections before making your selection.

☒ Life sciences ☐ Behavioural & social sciences ☐ Ecological, evolutionary & environmental sciences

For a reference copy of the document with all sections, see nature.com/documents/nr-reporting-summary-flat.pdf

Life sciences study design

All studies must disclose on these points even when the disclosure is negative.

Sample size

The total estimated sample size for the proposed intention-to treat, parallel-group, multicenter, randomized, controlled trial is 480 subjects. The primary endpoints are Visual Analog Scale (VAS) pain score and the pain subsection of Knee Injury and Osteoarthritis Outcomes (KOOS). The sample size calculations are based on improvements (from baseline to 1-year) in VAS pain score and total KOOS. KOOS total score was used as a proxy and should ensure the study is amply powered.
Considering a 10-point scale, assuming a decline of 1 point on average in pain in the Control arm (corticosteroids) and a decline on average of 2.5 points in a treatment arm (mesenchymal stem cells) and an estimated standard deviation on change of 3.5, the proposed sample sizes (n=121 subjects per group or 484 total subjects) will provide 91% power to detect a difference on change of 1.5 points at the two-sided 5% significance level if the true difference between treatment arms is 1.5 points (two-sided two-sample equal variance t-test).
Assuming an increase of 10 points on average in total KOOS in the Control arm (corticosteroids) and an increase on average of 20 points in a treatment arm (mesenchymal stem cells) and an estimated standard deviation on change of 20, the proposed sample sizes (n=121 subjects per group or 484 total subjects) will provide 97% power to detect a difference on change of 10 points at the two-sided 5% significance level if the true difference between treatment arms is 10 points (two-sided two-sample equal variance t-test).
Each of the four participating sites will accrue 30 subjects to each of the 4 treatment arms. Cohort retention is expected to be 90% at 1-year.

Data exclusions

The Per-Protocol Population (PP) includes subjects in the ITT population without any major protocol deviations. A major protocol deviation is a deviation that may significantly impact the completeness, accuracy, and/or reliability of the trial data; that may significantly affect a subject's rights, safety, or well-being (ICH E3 R1 Guidelines 2013).

Replication

The primary analyses of the data were performed according to subjects' original treatment assignment (i.e., intention-to-treat analyses) regardless of their compliance and the inclusion of all data from all subjects randomized in the final analysis. Sensitivity analyses were used to ensure our results were robust. Observed case (secondary) and multiple imputation methods (primary) were used for the primary ITT efficacy analysis

Randomization

The study includes a parallel design using a blocked central randomization scheme of 1:1:1:1. Subjects, who have provided written informed consent, will be randomized to one of the following treatment arms: Arm 1 includes randomization to bone marrow derived MSCs versus corticosteroid injection.
Arm 2 includes randomization to adipose derived MSCs versus corticosteroid injection.
Arm 3 includes randomization to umbilical cord tissue MSC's versus corticosteroid injection. Four hundred eighty subjects will be randomly assigned to treatments ensuring the trial is single-blind with a 1:1:1:1 allocation ratio across the four treatment arms. One hundred twenty subjects will be randomized at each of 4 clinical sites.
Arm 1: Forty subjects will be randomized with a 3:1 allocation ratio (bone marrow derived MSCs versus corticosteroid injection; 30:10). This same implementation plan will be used to randomize 40 subjects to Arm 2 (adipose derived MSCs versus corticosteroid injection) with a 3:1 allocation ratio and to randomize 40 subjects to Arm 3 (umbilical cord tissue MSC's versus corticosteroid injection) with a 3:1 allocation ratio.

Blinding

As a single-blinded study, the site principal investigators were not required to be blinded, however subjects were blinded to their injection. The blinding was implemented by limiting visualization of the syringe contents with opaque covering.

Reporting for specific materials, systems and methods

We require information from authors about some types of materials, experimental systems and methods used in many studies. Here, indicate whether each material, system or method listed is relevant to your study. If you are not sure if a list item applies to your research, read the appropriate section before selecting a response.

Materials & experimental systems

n/a	Involved in the study
☒	☐ Antibodies
☒	☐ Eukaryotic cell lines
☒	☐ Palaeontology and archaeology
☒	☐ Animals and other organisms
☐	☒ Clinical data
☒	☐ Dual use research of concern

Methods

n/a	Involved in the study
☒	☐ ChIP-seq
☒	☐ Flow cytometry
☐	☒ MRI-based neuroimaging

Clinical data

Policy information about clinical studies

All manuscripts should comply with the ICMJE guidelines for publication of clinical research and a completed CONSORT checklist must be included with all submissions.

Clinical trial registration: ClinicalTrials.gov Identifier number is NCT03818737.

Study protocol: Full study protocol included with manuscript submission and in the public domain on the clinicaltrials.gov site

Data collection: 570 patients were screened to identify 480 eligible patients that were randomized at five clinical sites in five different states within the United States of America. The first subject was enrolled in March 2019 and last subject completed the study in June 2022.

Outcomes: The primary efficacy co-outcomes are: a change in the visual analog score (VAS) pain score and a change in the pain subsection of the knee injury and osteoarthritis outcome (KOOS) score. Secondary outcomes include change in total KOOS score, EuroQuality of Life (EQ5D 3L) and Patient-Reported Outcomes Measurement Information System (PROMIS 29).
Changes in MRI biomarkers of cartilage and joint health between the four treatment groups will be compared.

Magnetic resonance imaging

Experimental design

Design type: Absolute change in MRI Score from baseline to month 12 will be analyzed using an ANCOVA

Design specifications: site, treatment arm and baseline MRI Score as fixed effects; the p-values for the treatment comparison, estimates of the treatment difference and the 95% confidence interval of the difference will be generated from the ANCOVA model.

Behavioral performance measures: No behavioral performance measures were taken

Acquisition

Imaging type(s): Structural (morphologic sequences) and biochemical (T2 map sequence)

Field strength: 3-Tesla

Sequence & imaging parameters: For structural MRI, we used 2D fast spin echo in axial, coronal, and sagittal planes, without and with fat suppression. For biochemical MRI, we used 2D fast spin echo multi-echo sequence to determine T2 relaxation values in cartilage and menisci.

Area of acquisition: whole knee joint

Diffusion MRI: ☐ Used ☒ Not used

Preprocessing

Preprocessing software: No preprocessing software was used

Normalization: No normalization was performed

Normalization template: No normalization template was used

Noise and artifact removal: No Noise and artifact removal was performed

Volume censoring: No volume censoring was performed

Statistical modeling & inference

Model type and settings: For T2 mapping, we used a mono-exponential T2 decay model

Effect(s) tested: The Difference of Absolute Change in MRI Cartilage Loss Extent Score from Baseline between each treatment group (3) and the CSI (control) group.

Specify type of analysis: ☐ Whole brain ☒ ROI-based ☐ Both

Anatomical location(s): manual segmentation was used

Statistic type for inference (See Eklund et al. 2016): Score as response variable and site, treatment arm and baseline MRI score as fixed effects.

Correction: Hochberg adjustment method is used: order the p-values from high to low and compare the largest p value to 0.05, the middle p value
to 0.05/2, and the smallest p value to 0.05/3. This is used to compare LS Means of the Difference of Absolute Change in MRI Cartilage Loss Extent Score from Baseline between each treatment group (3) and the CSI (control) group.

Models & analysis

n/a	Involved in the study	
☒	☐	Functional and/or effective connectivity
☒	☐	Graph analysis
☐	☒	Multivariate modeling or predictive analysis

Multivariate modeling and predictive analysis:
Ancova model with MRI Score at each visit as dependent variable
No extraction or dimension reduction
No training
No evaluation
